# Carbon monoxide‐triggered near‐infrared photoacoustic‐fluorescent integrated visualization tool for auxiliary diagnosis of heart failure and evaluation of reversal drug efficacy

**DOI:** 10.1002/smo2.70044

**Published:** 2026-04-17

**Authors:** Hongze He, Jingrui Yang, Yu Wu, Ping Lin, Tianxiao Lai, Junjie Jia, Jiangfeng Li, Weiying Lin

**Affiliations:** ^1^ Institute of Optical Materials and Chemical Biology Guangxi Key Laboratory of Electrochemical Energy Materials School of Chemistry and Chemical Engineering Guangxi Key Laboratory of Special Biomedicine School of Medicine Guangxi University Nanning Guangxi China

**Keywords:** carbon monoxide, fluorescent imaging, heart failure, photoacoustic imaging, reversal of heart failure

## Abstract

Heart failure (HF), a major global health challenge with high morbidity and mortality, lacks effective biomarkers and non‐invasive tools for early diagnosis and therapy evaluation. Endogenous carbon monoxide (CO), a key biological signaling molecule, is closely linked to cardiovascular diseases such as HF when dysregulated. Integrating near‐infrared (NIR) fluorescence (FL) imaging with photoacoustic (PA) imaging offers a powerful approach, as the 2D FL data can validate and supplement the 3D PA information for a more holistic disease assessment. Herein, we developed **CS‐CO**, an activatable dual‐mode NIR probe for the selective monitoring of CO fluctuations in HF. Density functional theory (DFT) calculations validated the design, elucidating the signal turn‐on mechanism upon CO binding. The metal‐free **CS‐CO** probe selectively detects CO, resulting in activated NIR absorption (710 nm) and emission (742 nm). Consequently, its excellent NIR PA and FL performance allowed for visualizing both exogenous and endogenous CO in cells, and for 2D FL and 3D PA imaging in mice. Crucially, using this dual‐mode imaging approach, **CS‐CO** successfully revealed dynamic CO level changes during both HF progression and drug‐induced reversal for the first time. This study provides a pioneering strategy for early HF diagnosis and drug efficacy evaluation, with significant potential clinical impact.

## Graphical abstract

An activatable metal‐free probe **CS‐CO** enables the first non‐invasive photoacoustic/fluorescence visualization of carbon monoxide (CO) fluctuations during heart failure and its reversal, linking CO levels directly to disease status.

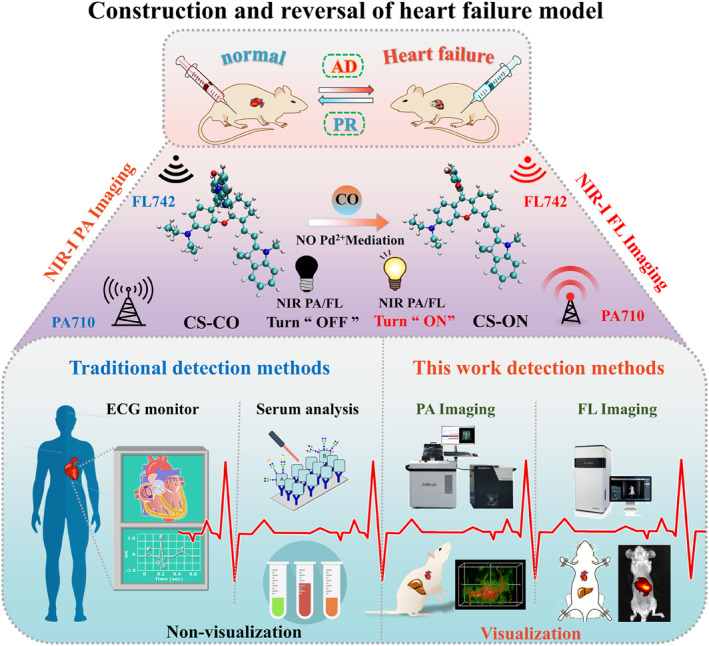

## 1 INTRODUCTION

Cardiovascular disease is the world's biggest medical burden, and it is increasing every year. Heart failure (HF), an important factor in cardiovascular mortality, is a syndrome of widespread prevalence, reduced quality of life, high treatment costs, and complex treatment options.[^1^, ^2^, ^3^] Failure to diagnose HF in time may lead to missing the best opportunity for treatment, aggravating the condition and even endangering the life of the patients. In addition, more and more researchers are currently working on drugs to reverse HF, which is of great importance for promoting human health and prolonging human lifespan. Current standard diagnostic approaches for HF primarily rely on clinical symptoms, physical signs, and auxiliary examinations.[^4^, ^5^] Nevertheless, these methods are often cumbersome, unable to yield in vivo measurement data, and lack real‐time monitoring capabilities.^[6]^ Therefore, there is a pressing demand to establish robust methods for the early diagnosis of HF, to elucidate its underlying pathogenesis, and to accurately assess the efficacy of novel reversal drugs.

Exogenous carbon monoxide (CO) is classically regarded as a toxic molecule and organism asphyxiant.^[7]^ Nevertheless, many recent studies have increasingly shown that endogenous CO is an important molecular signal transmitter for many normal physiological processes and the restoration of homeostasis in pathophysiological states.[^8^, ^9^] Known effects of endogenous CO on the cardiovascular system include modulation of autonomic nervous system input to both the primary pacemaker and the working myocardium, vasodilation, and changes in heart rate and myocardial contractility.[^10^, ^11^] Abnormal levels of endogenous CO may cause HF.[^12^, ^13^, ^14^] Therefore, the development of a reliable and powerful tool for specifically detecting CO concentration fluctuations in HF is of great significance for the exploration and diagnosis of HF‐related diseases and for the evaluation of drugs to reverse HF.

Within the broad spectrum of non‐invasive biomedical imaging technologies, photoacoustic (PA) imaging stands out by integrating the inherent merits of photoexcitation and acoustic detection. This distinctive integration empowers PA imaging to produce three‐dimensional (3D) images—capable of precisely delineating the spatial position, morphological features, dimensional parameters, and tissue boundaries—while simultaneously achieving high resolution and excellent imaging depth.^[15]^ Fluorescence (FL) imaging is a cornerstone technique in clinical diagnosis, prized for its high sensitivity, superior surface resolution, capacity for high‐throughput multi‐target imaging, real‐time capability, and excellent contrast.[^16^, ^17^, ^18^, ^19^, ^20^, ^21^, ^22^, ^23^, ^24^, ^25^] Complementarily, the utilization of near‐infrared (NIR) window dyes for developing PA or FL probes offers significant advantages, including an enhanced signal‐to‐noise ratio, increased permissible laser exposure, deeper tissue penetration, and minimized background interference and photo damage.[^26^, ^27^, ^28^, ^29^] Therefore, the integration of NIR FL and NIR PA imaging creates a powerful synergistic platform, where the detailed 2D FL data can verify and enrich the 3D structural information from PA, thereby greatly facilitating a comprehensive multi‐scale assessment of complex diseases such as HF. In previous studies, most probes required the presence of the transition metal Pd^2+^ to recognize CO.[^30^, ^31^, ^32^, ^33^] However, high concentrations of Pd^2+^ have serious toxicity to biological systems, and can even cause asthma, conjunctivitis and cancer in severe cases. These potential hazards limit the further application of these probes in vivo.[^30^, ^34^] Toward this end, it is crucial to develop a novel transition metal‐free activatable NIR PA and FL probe to diagnose HF and evaluate the efficacy of drugs to reverse HF through fluctuations in CO levels.

In this work, a novel activatable NIR PA/FL dual‐modal probe for CO detection was elaborately designed and synthesized, which exhibits a turn‐on type specific response toward elevated CO concentrations. The structural rationality of this NIR PA/FL dual‐modal probe was verified through density functional theory (DFT) calculations. Notably, the probe demonstrates exceptional sensitivity and remarkable selectivity for CO. Specifically, the NIR PA and FL signals of the developed probe can be specifically triggered by CO in both cellular and murine models. This unique property enables the dual‐modal probe to eliminate interference from endogenous biological optical signals during the response process, thereby achieving accurate CO recognition with high contrast and an excellent signal‐to‐noise ratio (SNR). Of great significance, this robust visualization tool was first applied for the diagnosis and investigation of HF, as well as the evaluation of therapeutic efficacy for reversing HF via NIR PA and FL imaging techniques. Through NIR PA imaging, we successfully visualized the upregulation of CO levels in adriamycin (AD)‐induced HF mice and the downregulation of CO levels in probucol‐treated mice with reversed HF from a three‐dimensional (3D) spatial perspective.

## 2 RESULTS AND DISCUSSION

### 2.1 Design of the NIR PA/FL dual‐modal probe **CS‐CO**

**SCHEME 1:**
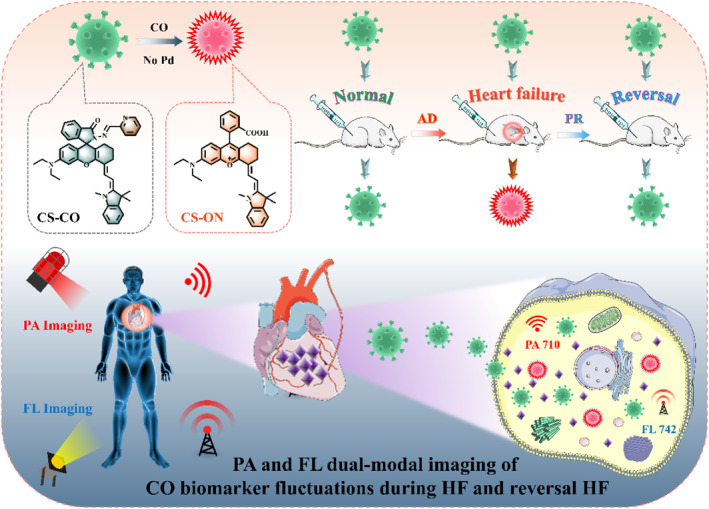
Photoacoustic and fluorescence dual‐modal imaging of carbon monoxide biomarker fluctuations during HF and reversal HF. HF, heart failure.

To enable the specific detection of CO fluctuations in living organisms by NIR PA and FL dual‐modality imaging, the selection of a dye with both NIR absorption and emission is crucial for a usable probe in complex biological systems. The often applied NIR dyes are mainly limited to a few traditional NIR dyes, such as cyanines, squaraines, and boron dipyrromethene derivatives. However, these dyes generally have no optically available tunable groups, rendering them often favorable as simple PA or FL tags for biospecies but not as platforms for developing target‐sensitive PA or FL probes. In comparison, hemicyanine dyes with benzoic acid groups may have excellent potential in the field of PA and FL bioimaging due to their strong optics ON‐OFF switching mechanism like rhodamine dyes and simultaneous optical absorption and FL emission in the NIR region.^[35]^ Meanwhile, hemicyanine dyes bearing benzoic acid groups have remarkable chemical stability,^[36]^ which is beneficial for long‐term and stable PA and FL imaging in complex living systems. On the other hand, conventional CO probes that require Pd^2+^ as a substrate will lead to dilution of Pd^2+^ when the probe and Pd^2+^ are co‐injected in vivo, thus seriously affecting the response of the probe to CO. In other words, the dilution of Pd^2+^ would reduce the sensitivity of the probe to CO during the disease process. Simultaneously, if the concentration of Pd^2+^ is increased, it will bring significant toxicity risk. To enable specific detection of CO in the absence of Pd^2+^, 2‐(hydrazonomethyl)‐pyridine was used as the recognition site for CO.^[37]^ Toward this end, in this work, we used the hemicyanine dye bearing benzoic acid groups as the optical scaffold and combined with 2‐(hydrazonomethyl)‐pyridine as the recognition group for specific detection of CO. The possible mode of the response of PA and FL dual‐modal probe **CS‐CO** to CO during HF and reversal of HF is shown in Scheme 1 and Figure S1.

### 2.2 DFT calculation of NIR probe **CS‐CO**

**FIGURE 1:**
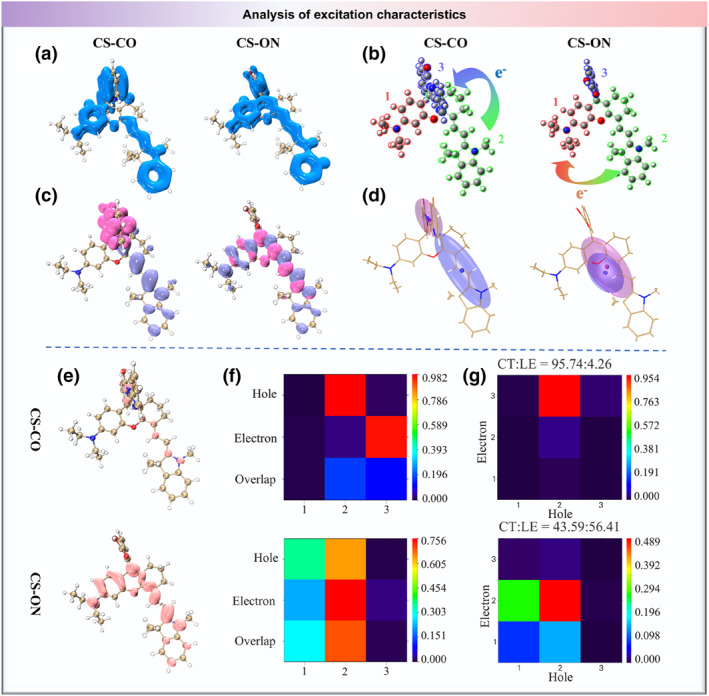
Excitation characterization of **CS‐CO** and **CS‐ON** *S*_0_ → *S*_1_. (a) LOL‐π isosurface map, (b) fragments and electron transfer pathway, (c) hole‐electron distribution (hole: blue, electron: purple), (d) hole‐electron centroid, (e) hole‐electron overlap function (Sr), (f) contribution heat map of fragment to hole, electron, and their overlap, and (g) interfragment charge transition density matrix heat map.

First, DFT and time‐dependent DFT (TD‐DFT) theoretical calculations were performed on **CS‐CO** and **CS‐ON** to preliminarily understand the photophysical properties of the FL off/on mechanism of the closed‐ring form of **CS‐CO** and the open‐ring form of **CS‐ON**. We first studied the structural differences between **CS‐CO** and **CS‐ON** (Figure S2). The core structures of both have good planarity, but the LOL‐π isosurface map (Figure 1a) clearly shows that compared with **CS‐ON**, the internal π electron conjugation path of **CS‐CO** is cut off by the carbon atoms of the cyclospiro ring, and a large conjugated system cannot be formed. The transition from the ring‐closed form of **CS‐CO** to the ring‐open form of **CS‐ON** involves the destruction and formation of the conjugated system between molecular fragments, which seems to correspond to the mechanism of FL switching from off to on.^[38]^ Therefore, we defined three structural fragments (Figure 1b), and by analyzing the hole‐electron and inter‐fragment charge transfer of *S*_0_ → *S*_1_ (Figure 1c–g) and *S*_1_ → *S*_0_ (Figure S3 and Tables S1–S3) of **CS‐CO** and **CS‐ON**, the two are obviously different: the vertical absorption (*S*_0_ → *S*_1_) of **CS‐CO** excites the CT excitation of non‐planar conjugation. The holes and electrons have a large degree of separation, and the electrons mainly flow from fragment 2 to fragment 3; the vertical absorption (*S*_0_ → *S*_1_) of **CS‐ON** excites the hybridized local and charge‐transfer excitation (HLCT) within the planar conjugation, and the electrons mainly flow from fragment 2 to fragment 1. The truncation of the **CS** dye parent conjugation path caused by **CS‐CO** and **CS‐ON** by changeable π‐conjugated system is the key to the FL on/off regulation. This conclusion is also confirmed by the reduction of the HOMO‐LUMO gap (Figure S4 and Table S4). At the same time, the reduced HOMO‐LUMO gap may also lead to more excitation energy consumption through non‐radiative transitions at *S*_1_ state.

**FIGURE 2:**
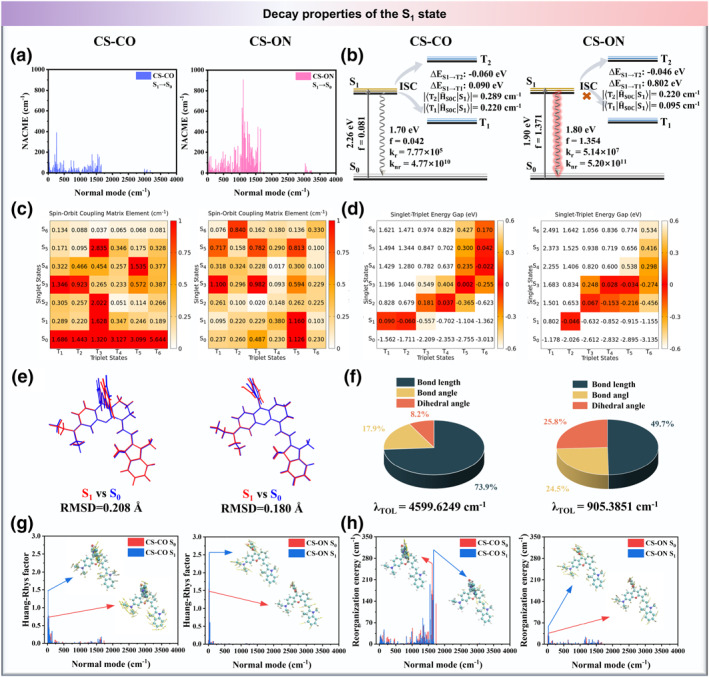
Decay properties of **CS‐CO** and **CS‐O** **N**. (a) Non‐adiabatic coupling matrix elements of each normal mode, (b) photophysical process, (c) Spin‐orbit coupling matrix element between *S*_n_ and *T*_m_, and (d) energy gap between *S*_n_ and *T*_m_. (e) Geometric comparison and root mean square deviation between *S*_0_ and *S*_1_, (f) contributions of bond length, bond angle and dihedral angle to the total reorganization energy. (g) Huang‐Rhys factor for *S*_1_ → *S*_0_. (h) Recombination energy for *S*_1_ → *S*_0_. (Representative normal modes are shown in illustrations.)

To further verify that **CS‐CO** has good potential for PA imaging and FL imaging in response to CO, we studied the non‐radiative transition process (*k*_nr_) and radiative transition process (*k*_r_) of **CS‐CO** and **CS‐ON** in the NIR region. As shown in Figure 2a, compared with the probe **CS‐CO**, the non‐adiabatic coupling of **CS‐ON** increased significantly, indicating that the ability of **CS‐ON** was greatly enhanced when undergoing the *k*_nr_ process. We further calculated the photophysical process of *S*_1_ and *S*_0_ of **CS‐CO** and **CS‐ON** (Figure 2b), and the difference in oscillator strength also revealed the mechanism of FL switching off and on for **CS‐CO** and **CS‐ON**. Specifically, the *k*_r_ of **CS‐CO** and **CS‐ON** in the NIR region were 7.77 × 10^5^ and 5.14 × 10^7^ s^−1^, respectively, an increase of 100 times; the *k*_nr_ were 4.77 × 10^10^ and 5.20 × 10^11^ s^−1^, respectively, an increase of 10 times. These results indicate that the significant improvement in *k*_r_ and *k*_nr_ of **CS‐ON** makes it possible to activate **CS‐CO** in NIR FL and PA imaging. Figure 2c,d show the spin‐orbit coupling matrix element and the singlet‐triplet energy gap diagram, which further reveals the possible intersystem crossing.[^39^, ^40^] The data analysis of the root mean square deviation of *S*_1_ and *S*_0_, the Huang‐Rhys factor, and the total reorganization energy (Figures 2e–h and S5) shows that the total reorganization energy of **CS‐CO** (4599.6249 cm^−1^) is greater than that of **CS‐ON** (905.3851 cm^−1^), indicating that a large amount of energy is consumed in the excited state (*S*_1_) of **CS‐CO** for non‐radiative decay. Based on the above theoretical analysis results, we are convinced that the design of the new activatable NIR PA and fluorescent dual‐mode CO probe **CS‐CO** is reasonable.

### 2.3 Photophysical properties of the NIR probe **CS‐CO**

**FIGURE 3:**
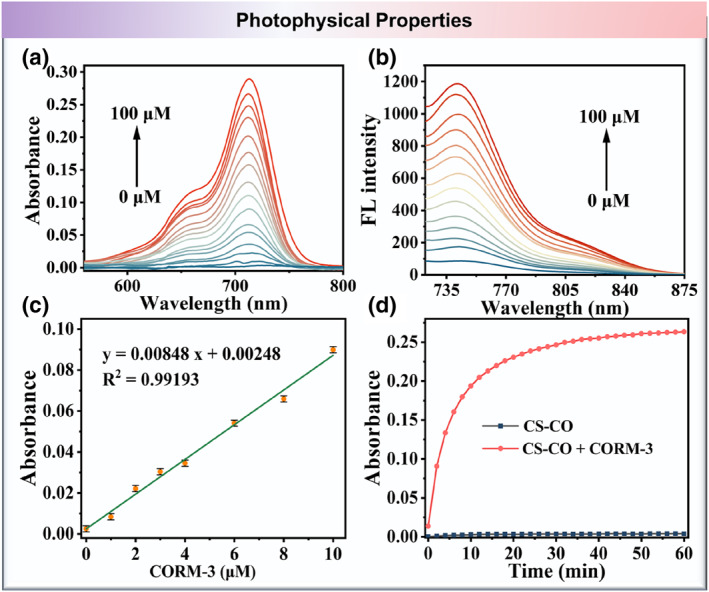
(a) Absorption spectra of **CS‐CO** (10 μM) after incubation with various concentrations (0–100 μM) of CORM‐3. (b) Fluorescence spectra of **CS‐CO** (10 μM) treated with various concentrations (0–100 μM) of CORM‐3. (c) Linear relationship between the absorption of **CS‐CO** and 100 μM CORM‐3 concentration. (d) Time response profiles of **CS‐CO** (10 μM) to CORM‐3 (100 μM).

Guided by the theoretical calculations, we subsequently investigated the optical response of **CS‐CO** toward CO. The probe **CS‐CO** was synthesized according to Scheme S1 to obtain an activatable agent sensitive to CO for NIR PA/FL dual‐modal imaging. Its structure was unambiguously confirmed by ^1^H nuclear magnetic resonance (NMR), ^13^C NMR, and high resolution mass spectrometry (HRMS) (see Figures S6–S8). We then evaluated the responsiveness of **CS‐CO** to CO in phosphate buffered saline (PBS) buffer (10 mM, pH 7.4). As shown in the UV–vis spectra (Figure 3a), the free probe **CS‐CO** exhibited no significant absorption at 710 nm. However, upon the addition of [Ru(CO)_3_Cl‐(glycinate)] (CORM‐3, a CO donor), a notable absorption peak emerged and intensified, reaching a maximum (a 52.4‐fold enhancement) upon reaction with 100 μM CORM‐3. Parallel experiments exploring the NIR FL response revealed that treating **CS‐CO** (10 μM) with increasing concentrations of CORM‐3 (0–100 μM) led to a gradual enhancement of the FL intensity at 742 nm (Figure 3b). A satisfactory linear relationship between the FL intensity and CO concentration (0–10 μM) was observed, with a calculated detection limit of approximately 0.59 μM (Figure 3c). To substantiate that these optical changes originated from the reaction between **CS‐CO** and CO, we performed HRMS analysis to identify the resultant reaction product. A new mass peak was fined at m/z 559.2958 (Figure S9), which is identical to the dye **CS‐ON** ([C_37_H_39_N_2_O_3_^+^]: 559.2955). These results imply that the 2‐(hydrazonomethyl)‐pyridine group of **CS‐CO** might be ring‐opened by CO under physiological conditions, leading to the conversion of **CS‐CO** to **CS‐ON**.

Kinetic studies indicated that the reaction reached completion within 30 min upon adding 100 μM CORM‐3 (Figure 3d). **CS‐CO** also showed robust temporal stability over 60 min (Figure S10). Most importantly, the selectivity assay (Figure S11) showed a dramatic NIR absorption increase at 710 nm only for CORM‐3, with other species causing negligible changes, confirming the excellent selectivity for CO. This response remained effective across a wide physiological pH range (Figure S12). In conclusion, the probe **CS‐CO** exhibits high specificity and sensitivity for CO in the NIR optical windows. These compelling in vitro results unmistakably confirm its potential for subsequent application in specific NIR PA and FL imaging of CO in vivo.

### 2.4 Visualization of exogenous and endogenous CO in cells with NIR probe **CS‐CO**

**FIGURE 4:**
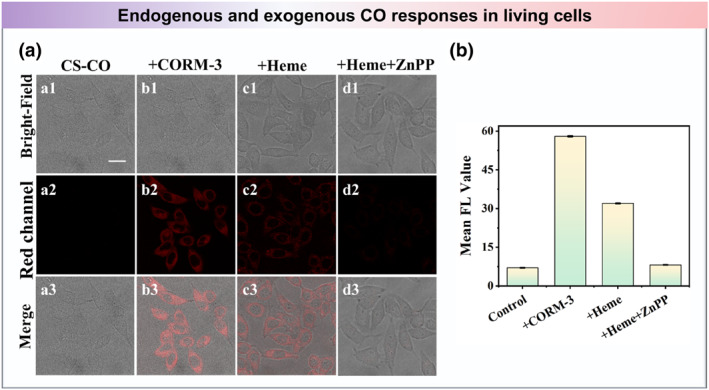
(a) Fluorescence images for HeLa cells. (a1–a3) Cells were incubated with **CS‐CO** (10 μM) for 1 h. (b1–b3) Cells were treated with **CS‐CO** (10 μM) and CORM‐3 (100 μM) for 1 h. (c1–c3) Cells were pretreated with heme (100 μM) for 12 h, and then incubated with **CS‐CO** (10 μM) for 1 h. (d1–d3) Cells were treated with 100 μM heme and 1 μg/mL ZnPP for 12 h, and then incubated with **CS‐CO** (10 μM) for 1 h. (b) Mean intensity in cells imaging. *λ*_ex_ = 670 nm, *λ*_em_ = 700–790 nm. Scale bar: 20 μm.

The 3‐(4,5)‐dimethylthiahiazo(‐z‐y1)‐3,5‐diphenytetrazolium bromide assay confirmed the favorable biocompatibility of **CS‐CO** with very low cytotoxicity (Figure S13), enabling subsequent cell imaging studies. To test its functionality in a biological context, we applied **CS‐CO** to detect exogenous CO in live HeLa cells. Cells incubated with **CS‐CO** alone showed minimal FL, whereas a robust turn‐on signal was observed in cells co‐treated with **CS‐CO** and CORM‐3 (Figure 4a). This response was quantified as an 8.2‐fold increase in NIR FL intensity (Figure 4b), definitively demonstrating the probe's ability to respond to CO in living cells. Finally, to expand its application to endogenous CO, which can be produced under heme‐induced oxidative stress.[^41^, ^42^] The efficacy of **CS‐CO** for detecting endogenous CO was confirmed in HeLa cells, where heme stimulation caused a 4.5‐fold FL increase. This signal was specifically suppressed by the HO‐1 inhibitor ZnPP,^[43]^ verifying its origin from intracellular CO. These results conclusively demonstrate that **CS‐CO** serves as a potent and specific tool for visualizing CO in biological systems.

### 2.5 NIR PA/FL dual‐mode CO imaging in mice

**FIGURE 5:**
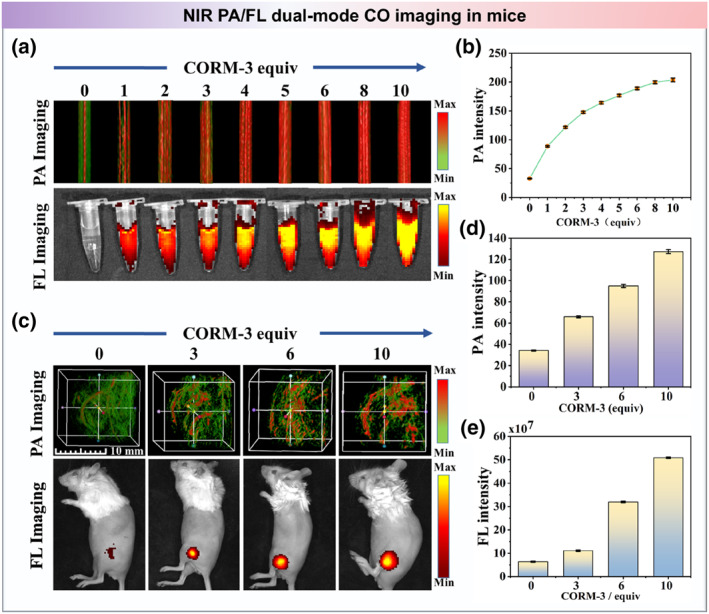
(a) The NIR PA and fluorescence imaging of **CS‐CO** (30 μM) after adding varying concentrations of CORM‐3. (b) PA signals intensity of **CS‐CO** after treatment with different concentrations of CORM‐3. (c) In vivo NIR PA and FL imaging of **CS‐CO** (30 μM) at various concentrations of carbon monoxide (CO) in mice. (d) Signals intensity values of in vivo NIR PA imaging of **CS‐CO** with different concentrations of CORM‐3 in mice. (e) Signals intensity values of in vivo NIR FL imaging of **CS‐CO** with various concentrations of CORM‐3 in mice. FL, fluorescence; NIR, near‐infrared; PA, photoacoustic.

The specific responsiveness of **CS‐CO** to CO was preliminarily evaluated by NIR PA and FL imaging in solution. The probe demonstrated a concentration‐dependent turn‐on response to CORM‐3 (0–10 equiv) in both modes (Figure 5a), with signal enhancements of 7.5‐fold (PA) and 6.1‐fold (FL) at the highest concentration (Figures 5a,b and S14). This performance definitively establishes the probe's strong potential for subsequent NIR PA and FL imaging in vivo.

Following successful in vitro tests, we assessed **CS‐CO** in live mice. The probe showed good biocompatibility, with all mouse groups displaying normal weight gain (Figure S15). In a subcutaneous imaging model, co‐injection of **CS‐CO** with increasing CORM‐3 equivalents (0, 3, 6, 10 equiv) (Figure 5c) produced a clear dose‐dependent turn‐on response in both NIR PA_710_ and FL_742_ signals, against a low background from the probe alone (Figure 5d,e). These results confirm **CS‐CO** as a capable, non‐invasive, dual‐mode imaging tool for CO detection in vivo.

### 2.6 NIR PA/FL dual‐mode imaging in the animal model of heart failure and reversal of heart failure

**FIGURE 6:**
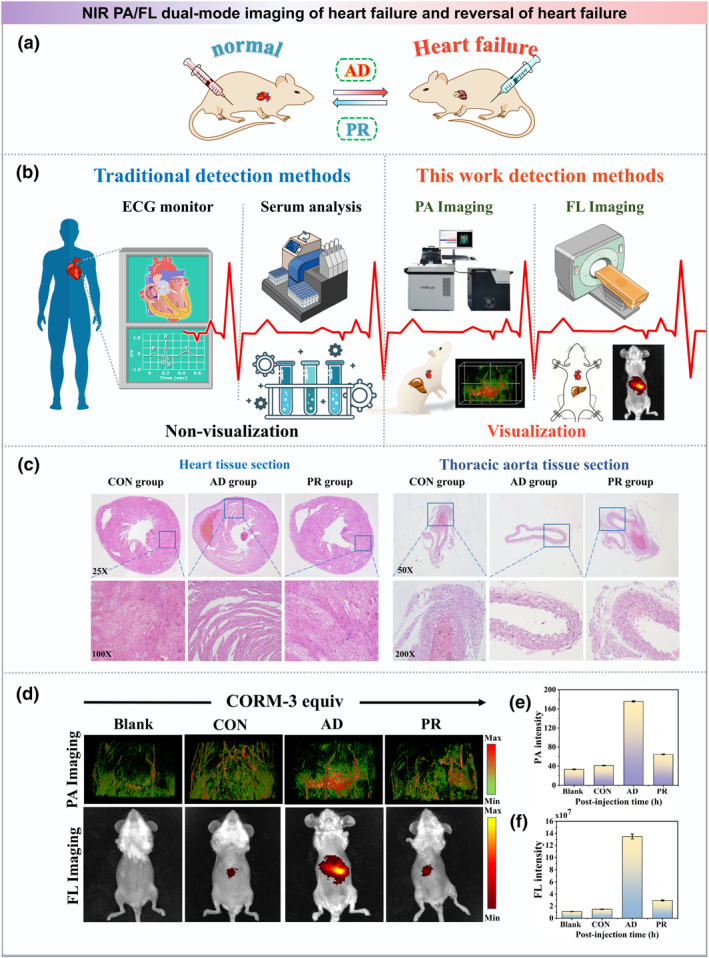
(a) Establishment process of HF and HF reversal in mice. (b) Imaging processes in HF and HF reversal mice. (c) H&E staining of heart and thoracic aorta. (d) NIR PA imaging of normal mice (CON group), HF mice (AD group) and HF reversal mice (PR group) after tail vein injection of **CS‐CO**. (e) Signals intensity values of NIR PA imaging of **CS‐CO** in normal mice (CON group), HF mice (AD group) and HF reversal mice (PR group). (f) Signal intensity values of NIR FL imaging of **CS‐CO** in normal mice (CON group), HF mice (AD group) and HF reversal mice (PR group). HF, heart failure; NIR, near‐infrared; PA, photoacoustic.

Altered CO metabolism is closely linked to the pathogenesis of HF,^[44]^ highlighting the need to monitor CO in disease models. We therefore investigated the imaging ability of **CS‐CO** in a classic AD‐induced mouse model of HF[^45^, ^46^] (Figure 6a,b). In addition, probucol (PR), an antioxidant and lipid‐lowering drug widely employed in clinical settings, has been shown to have preventive effects on HF. By augmenting the endogenous antioxidant system and regulating apoptosis and high lipid levels, probucol exerts a cardioprotective function and helps prevent the onset of HF.[^47^, ^48^] Thereby, we further applied probucol to treat AD‐induced HF. To initially study HF and evaluate the efficacy of probucol in reversing HF, we first established mice model of AD‐induced HF and probucol (PR) treatment of AD‐induced HF. We further examined the heart and thoracic aorta tissues of these model mice to gain a better understanding of the underlying processes involved. According to H&E staining (Figure 6c), we found that the adriamycin (AD) group had left ventricular myocyte hypertrophy and increased wall thickness, perivascular fibrosis, and cardiomyocyte disorder compared with the control (CON) group. In addition, we found that arterial wall hypertrophy, the number of vascular smooth muscle cells increased and the cells were disordered in the AD group. By sharp contrast, in the PR group, myocardial and perivascular fibrosis, left ventricular wall thickness, and disorganized smooth muscle cells were reduced after probucol treatment. Taken together, these results demonstrate that AD caused significant vascular and cardiac damage in mice, and that probucol reversed AD‐induced HF damage in mice.

As illustrated in Figure 6d, the PA and FL signal intensities in the AD‐induced HF group were significantly elevated compared to the CON and Blank groups. This result indicates an upregulation of CO concentration and a concomitant disruption of the oxidative stress balance in the HF model. Conversely, the signal intensities in the probucol‐treated (PR) group were markedly reduced relative to the AD group (Figure 6e,f). This suggests that probucol mitigates the AD‐induced metabolic dysfunction, leading to a subsequent decrease in CO levels. In summary, our non‐invasive visualization approach provides a direct tool for elucidating the role of CO in HF progression and therapeutic reversal. To our knowledge, this work represents the first instance where a PA/FL dual‐modal probe has been employed not only to visualize HF but also to evaluate the efficacy of a reversal drug non‐invasively. This pioneering strategy addresses a notable gap in the field and establishes a powerful and translatable platform for advancing human HF research.

## 3 CONCLUSION

In conclusion, we rationally designed **CS‐CO**, which is a metal‐free activatable NIR PA/FL probe to detect CO. Its design that conjugates a 2‐(hydrazonomethyl)‐pyridine group with the NIR dye **CS‐ON** was demonstrated by DFT recipes that demonstrated that **CS‐CO** had an adjustable π‐conjugated system, and increases in the NIR transition processes of *k*_nr_ and *k*_r_ following a response to CO, which led to the construction of a robust NIR absorption and emission D‐π‐A framework. The probe had sensitive and rapid dual‐mode turn‐on response at 710/742 nm. These capabilities were used to image CO in cells and live mice. It is an inventive implementation of **CS‐CO** in diagnosing HF and determining therapeutic reversal in vivo. With our dual‐modal imaging, increased CO was seen in the AD‐induced HF mice and decreased CO in probucol treated mice as an expression of 3D spatial data. Most importantly, the **CS‐CO** successfully identified deep‐tissue HF lesions using the high‐resolution 3D PA imaging method, which provides a new diagnostic approach as well as a potent instrument to investigate the role of CO in HF and expedite drug development.

## 4 EXPERIMENTAL SECTION

Detailed information on instruments, reagents, synthesis route of **CS‐CO**, structural characterization, computational details, optical studies, cell and animal experiments can be described in Supporting Information S1.

### 4.1 Theoretical calculation

Unless otherwise specified, all DFT and TDDFT calculations were performed using Gaussian 16 C.01^[49]^ and ORCA 6.0.1.[^50^, ^51^, ^52^, ^53^] Subsequently, the photophysical properties of **CS‐CO** and **CS‐ON** were computed using MOMAP 2024A.[^54^, ^55^, ^56^, ^57^, ^58^, ^59^] Additionally, Multiwfn 3.8 (dev),^[60]^ VMD 1.9.3,^[61]^ and GaussView 6 were employed for post‐processing wave function analysis and visualization. All atom coordinates after structural optimization are provided in Table S5–S8.

### 4.2 Optical studies

Unless otherwise noted, all experiments were performed in a 10 mM PBS buffer containing 50% MeOH solution at 37°C. A concentrated stock solution of **CS‐CO** was prepared at 2 mM concentration using dimethyl sulfoxide as the solvent.

### 4.3 The mouse model of heart failure and heart failure reversal

Mice were divided into three groups (*n* = 3/group) under identical conditions: the AD group received intraperitoneal AD (2.5 mg/kg) on days 1,3,5,7,9,11; the PR group received identical AD dosing plus probucol (10 mg/kg, i.p.) on alternate days (2,4,6,8,10,12); and the CON group received volume‐matched saline injections relative to the AD group. Details see Supporting Information S1.

## AUTHOR CONTRIBUTIONS

**Hongze He**: Conceptualization; writing—review and editing; visualization; methodology. **Jingrui Yang**: Visualization; formal analysis; writing—review and editing; software. **Yu Wu**: Data curation; investigation. **Ping Lin**: Visualization. **Tianxiao Lai**: Investigation. **Junjie Jia**: Validation. **Jiangfeng Li**: Conceptualization; writing—review and editing; methodology; funding acquisition. **Weiying Lin**: Resources; writing—review and editing; supervision; funding acquisition.

## CONFLICT OF INTEREST STATEMENT

The authors declare no conflicts of interest.

## ETHICS STATEMENT

All animal experiments were performed according to the guidelines of the Care and Use of Laboratory Animals formulated by the Ministry of Science and Technology of China. All experimental protocols were approved by the Institutional Animal Care and Use Committee of GuangXi University.

## Supporting information

Supporting Information S1

## Data Availability

The data that supports the findings of this study are available in the supplementary material of this article.
