# Supplementary material for: Carbon monoxide‐triggered near‐infrared photoacoustic‐fluorescent integrated visualization tool for auxiliary diagnosis of heart failure and evaluation of reversal drug efficacy
Source: Smart Mol. 2026 Apr 17;4(2):e70044. doi: 10.1002/smo2.70044 (PMC13317754; doi:10.1002/smo2.70044)
Supplement: Supplementary file 1 — Supporting Information S1 [file SMO2-4-e70044-s001.docx]

***Supporting Information for***

**Carbon monoxide-triggered NIR photoacoustic-fluorescent integrated visualization tool for auxiliary diagnosis of heart failure and evaluation of reversal drug efficacy**

*Hongze He, Jingrui Yang, Yu Wu, Ping Lin, Tianxiao Lai, Junjie Jia, Jiangfeng Li*, Weiying Lin **

*Institute of Optical Materials and Chemical Biology, Guangxi Key Laboratory of Electrochemical Energy Materials, School of Chemistry and Chemical Engineering, Guangxi Key Laboratory of Special Biomedicine, School of Medicine, Guangxi University, Nanning, Guangxi 530004, P. R. China.*

**Corresponding Author.*

*E-mail address: jiangfengli2022@163.com (J. Li), weiyinglin2013@163.com (W. Lin)*

**Table of contents**

Experimental Section S4

1. Experimental instruments and reagents S4

2. Synthesis and characterization of **CS-CO**. S4

3. General procedure for spectral measurement of **CS-CO** in response to CO. S5

4. The detection limit S5

5. Cell experiment S6

5.1. Culture and preparation of the HeLa cells S6

5.2. Cytotoxicity assay. S6

6. General procedure for PA and FL imaging of **CS-CO**. S7

7. Animal Experiments S7

7.1. PA/FL imaging of exogenous CO in mice S7

7.2. the mouse model of heart failure and heart failure reversal S7

7.3. In vivo PA/FL imaging of the mouse model of heart failure and heart failure reversal S8

Supplementary Figures S8

Figure S1 The possible response process of **CS-CO** for CO recognition. S8

Figure S2 Geometric structures and planarity of **CS-CO** and **CS-ON** in S_0_ state. S9

Figure S3 Excitation characterisation of **CS-CO** and **CS-ON** (S_1_→S_0_). S10

Figure S4 The Front molecular orbitals and energy gaps of **CS-CO** and **CS-ON**. S11

Figure S5 The representative normal modes of **CS-CO** and **CS-ON** in S_0_ and S_1_ states and their Huang-Rhys factors and reorganization energies. S12

Figure S6 The ^1^H NMR of **CS-CO** in CDCl_3_. S13

Figure S7 The ^13^C NMR of **CS-CO** in CDCl_3_. S13

Figure S8 ESI-MS spectrum of **CS-CO**. S14

Figure S9 HRMS (ESI) spectra of probe **CS-CO** and CORM-3 after reaction. S14

Figure S10 Photostability of **CS-CO** (10 μM) in the presence or absence of CORM-3 (100 μM). S15

Figure S11 Absorption intensity of **CS-CO** (10 μM) after addition of various analytes. S15

Figure S12 Absorption intensity changes of **CS-CO** at different pH values in the absence or presence of CORM-3. S16

Figure S13 Survival rate of HeLa cells after 24 h of incubation with different concentrations of **CS-CO** by MTT assays. S16

Figure S14 FL signal intensity of **CS-CO** after incubation with various concentrations of CORM-3. S16

Figure S15 Body weight change of the mice during 16 days following the intravenous injection of PBS (control group) and **CS-CO** (100 μM) into the tail vein of the mice. S17

Supplementary table S18

Table S1 Basic parameters of hole-electron analysis. S18

Table S2 Percentage contribution of each fragment to hole and electron. S19

Table S3 Net amount (e) of electron transfer between fragments. S20

Table S4 The orbital transition type, transition orbital contribution and π component of **CS-CO** and **CS-ON** under the transition (S_0_→S_1_). S20

Optimized atomic coordinate information S21

Table S5 Cartesian coordinates (Å) obtained after optimising **CS-CO** S_0_. S21

Table S6 Cartesian coordinates (Å) obtained after optimising **CS-ON** S_0_. S24

Table S7 Cartesian coordinates (Å) obtained after optimising **CS-CO** S_1_. S28

Table S8 Cartesian coordinates (Å) obtained after optimising **CS-ON** S_1_. S31

References S34

# Experimental Section

## Experimental instruments and reagents

Common reagents or materials were obtained from commercial suppliers without further purification except as otherwise noted. UV-vis absorption spectra were obtained on a Shimadzu UV-2700 spectrophotometer (Japan), and fluorescence spectra were measured on a HITACHI F4700 fluorescence spectrophotometer (Japan). The fluorescence imaging of cells were performed with Leica TCS SP8 CARS confocal microscope (Germany). The NIR fluorescence imaging of the mice was performed with a Small Animal In Vivo Imaging System (IVIS Lumina Series III). The photoacoustic imaging of mice and solutions were performed with the LOIS 3D imaging system (TomoWave Laboratories, and USA). ^1^H and ^13^C NMR spectra were measured on a Bruker Avance III HD 600 digital NMR spectrometer (Germany), using tetramethylsilane (TMS) as internal reference. High resolution mass spectrometric (HRMS) analyses were measured on Waters UPLC G2-XS Qtof (USA). TLC analysis was carried out on silica gel plates and column chromatography was conducted over silica gel (mesh 200-300), both of which were purchased from the Qingdao Ocean Chemicals. All aqueous solutions were prepared with ultrapure water obtained from a Milli-Q water purification system (18.2 MΩ cm).


## Synthesis and characterization of CS-CO.

**Scheme S1**. Synthetic route of the probe **CS-CO**.

**Synthesis of Compound** **CS-NH_2_.** The compound **CS-ON** was synthesized according to the previously literature method. ^[1]^ The raw material **CS-ON** (659.18 mg, 1 mmol), hydrazine hydrate (0.66 mL, 10 mmol) and BOP reagent (450 mg, 1 mmol) were added to a 25 mL round bottom flask containing 10 mL of dichloromethane. The solution was stirred at room temperature for 4.5 hours. After initial purification via chromatography (silica gel, DCM: CH_3_OH = 50: 1), the obtained yellow solid product was used directly for next reaction.

**Synthesis of Probe CS-CO.** Compound **CS-NH_2_** (57.23 mg, 0.1 mmol) and 2-pyridinecarboxaldehyde (53.52 mg, 0.5 mmol) were dissolved by 5 mL anhydrous methanol. The mixed solution was stirred for 5 h at room temperature. The solvent was removed by rotating evaporation. Then the yellow solid product was obtained after chromatography (silica gel, CH_2_Cl_2_: CH_3_OH = 10:1). Yield: 34.39 mg (52%).^1^H NMR (600 MHz, Chloroform-*d*) δ 8.62 (s, 1H), 8.53 (d, *J* = 4.3 Hz, 1H), 8.12 (d, *J* = 8.0 Hz, 1H), 7.99 (d, *J* = 7.5 Hz, 1H), 7.66 (t, *J* = 7.6 Hz, 1H), 7.56 – 7.53 (m, 2H), 7.47 (t, *J* = 7.4 Hz, 1H), 7.25 (d, *J* = 7.6 Hz, 1H), 7.20 (t, *J* = 7.7 Hz, 4H), 6.88 (t, *J* = 7.1 Hz, 1H), 6.64 (d, *J* = 7.6 Hz, 1H), 6.53 (d, *J* = 8.8 Hz, 1H), 6.42 (s, 1H), 6.27 (d, *J* = 7.2 Hz, 1H), 5.40 (d, *J* = 12.6 Hz, 1H), 3.35 (q, *J* = 6.9 Hz, 4H), 3.17 (s, 3H), 2.51 (s, 2H), 1.79 (d, *J* = 17.3 Hz, 6H), 1.42 (d, *J* = 28.3 Hz, 2H), 1.32 (d, *J* = 5.9 Hz, 2H), 1.19 (t, *J* = 7.0 Hz, 6H). ^13^C NMR (151 MHz, Chloroform-*d*) δ 165.69 , 157.73 , 154.67 , 152.02 , 149.02 , 148.89 , 147.44 , 145.39 , 138.92 , 136.19 , 133.60 , 128.63 , 127.68 , 127.15 , 123.66 , 123.48 , 123.35 , 121.55 , 120.75 , 119.67 , 119.28 , 108.35 , 105.68 , 104.27 , 98.38 , 92.05 , 67.83 , 45.50 , 44.26 , 31.65 , 31.60 , 29.71 , 29.67 , 29.13 , 28.43 , 25.23 , 22.84 , 22.67 , 22.14 , 14.14 , 12.63. HRMS (ESI): m/z calculated for C_43_H_43_N_5_O_2_ [M+H]^+^ 662.3490, found: 662.3487


## General procedure for spectral measurement of CS-CO in response to CO.

The stock solution of 2 mM the probe was prepared freshly in dry DMSO. For CO response experiments, absorption and emission spectroscopy were monitored after mixing PBS solution (10 mM, pH 7.4, containing 50% MeOH).

## The detection limit

According to the absorption titration curve of **CS-CO** in the presence of different concentrations of CORM-3, the detection limit can be calculated by the following quation (1):

Detection limit = 3σ/k (1)

Here, σ represents the standard deviation of the blank measurements, and k represents the slope of the curve of absorption intensity as a function of CORM-3 concentrations. The absorption spectrum of the blank sample was measured eleven times to calculate the standard deviation.

## Cell experiment

### Culture and preparation of the HeLa cells

The HeLa cells cultured in DMEM (Dulbecco’s modified Eagle's medium) supplemented with 10% FBS (fetal bovine serum) in an atmosphere of 5% CO_2_ and 95% air at 37 ℃. Before the experiments, seed the HeLa cells in 35-mm glass-bottomed dishes at a density of 2×10^5^ cells per dish in 2 mL of culture medium and incubate them inside an incubator containing 5% CO_2_ and 95% air at 37 ℃. Incubate the cells for 24 h. Cells will attach to the glass surface during this time.


### Cytotoxicity assay.

In this work, the cytotoxicity of **CS-CO** in living HeLa cells was investigated by MTT assay. HeLa cells were seeded at a density of 50000 cells/mL in a 96-well micro-assay culture plate and growth for 24 h in a 5% CO_2_/95% air incubator. The cell Dulbecco’s Modified Eagle Medium (DMEM) culture medium of each well was then replaced with the fresh medium containing increasing concentration of **CS-CO**, i.e., 0, 10, 20, 30, 40, and 50 µM. The wells with the cell culture media only were employed as the blank. After incubation in a 5% CO_2_/95% air incubator for 24 h, cell culture medium was removed and the cells were washed three times with PBS. Then, 100 μL of 0.5 mg/mL MTT solution in PBS was added to each well, and the cells were incubated for another 4 h. The excess MTT solution was then carefully removed from each well, and the formed formazan was dissolved in 100 μL of DMSO. The optical density of each well was measured at a wavelength of 490 nm using a microplate reader (Bio-Tek, USA). The results from the five individual experiments were averaged. The following formula was used to calculate the viability of cell growth: Viability = (mean of absorbance value of treatment group – blank) / (mean absorbance value of control – blank) × 100%. All of the measurements were performed five times and the values are presented as the mean ± SD.

## General procedure for PA and FL imaging of CS-CO.

In PA imaging, the excitation wavelengths used are **CS-CO** (710 nm). The excitation wavelengths used are **CS-CO** (700 nm), the collection wavelength ranges used are **CS-CO** (780 nm).

## Animal Experiments

The animals were purchased from the Experimental Animal Center of the Guangxi Medical University (Nanning, China). All animal experiments were reviewed and approved by the Animal Care and Experiment Committee of Guangxi University (protocol number: Gxu-2021-115).

### PA/FL imaging of exogenous CO in mice

The mice (4–5 weeks) were selected for in vivo imaging of exogenous CO. The probe solution (**CS-CO**, 0.1 mM each) was subcutaneously injected into the mice, and then different concentrations of CORM-3 (0, 3, 6, and 10 equiv) were injected into the same site. All of the mice were anesthetized with 2% isoflurane before imaging, and then the PA and FL imaging were recorded.

### the mouse model of heart failure and heart failure reversal

The mice (4–5 weeks) were purchased from School of Pharmaceutical Sciences, Guangxi Medical University and the mice were kindly kept during the experiments. All mice were divided into three groups and each with three raised in the same environment. As follows: the AD group (adriamycin-treated), the PR group (adriamycin plus probucol-treated), and the CON group (saline-treated, for control). Mice of the AD and PR groups were injected intraperitoneally with adriamycin (2.5 mg/kg) for different times (1, 3, 5, 7, 9, and 11 days). Then, mice of the PR group were also administered intraperitoneally with probucol (10 mg/kg) on alternate days (2, 4, 6, 8, 10, and 12 days). Mice of the CON group were administered saline intraperitoneally with an equal volume of the ADR group. After 12 days, the heart and the thoracic aorta were immediately removed and rinsed by saline. These biological tissues were further prepared into paraffin sections (stained with hematoxylin and eosin) and frozen sections (stained with 10 μM/L BCO for 30 min). Fluorescence images were observed by fluorescence microscopy (Leica Imaging Systems Ltd).

### In vivo PA/FL imaging of the mouse model of heart failure and heart failure reversal

The AD group mice, PR group mice, CON group mice and Balnk group were used for the following experiments (n = 3 mice for quantitative analyses of all imaging modalities). **CS-CO** (0.1 mM) was injected into the tail vein of AD group mice, PR group mice and CON group mice, respectively. Saline (0.1 mM) was injected into the tail vein of the Blank group mice, respectively. All of the mice were anesthetized with 2% isoflurane, and then PA and FL images were recorded. For fluorescence imaging, it was performed with a Small Animal In Vivo Imaging System with excitation at 700 nm. For PA imaging, it was performed with the same mice as FL imaging using a 3D PA imaging system (LOIS-3D, TomoWave Laboratories, USA). The PA data were acquired at 710 nm excitation.

# Supplementary Figures


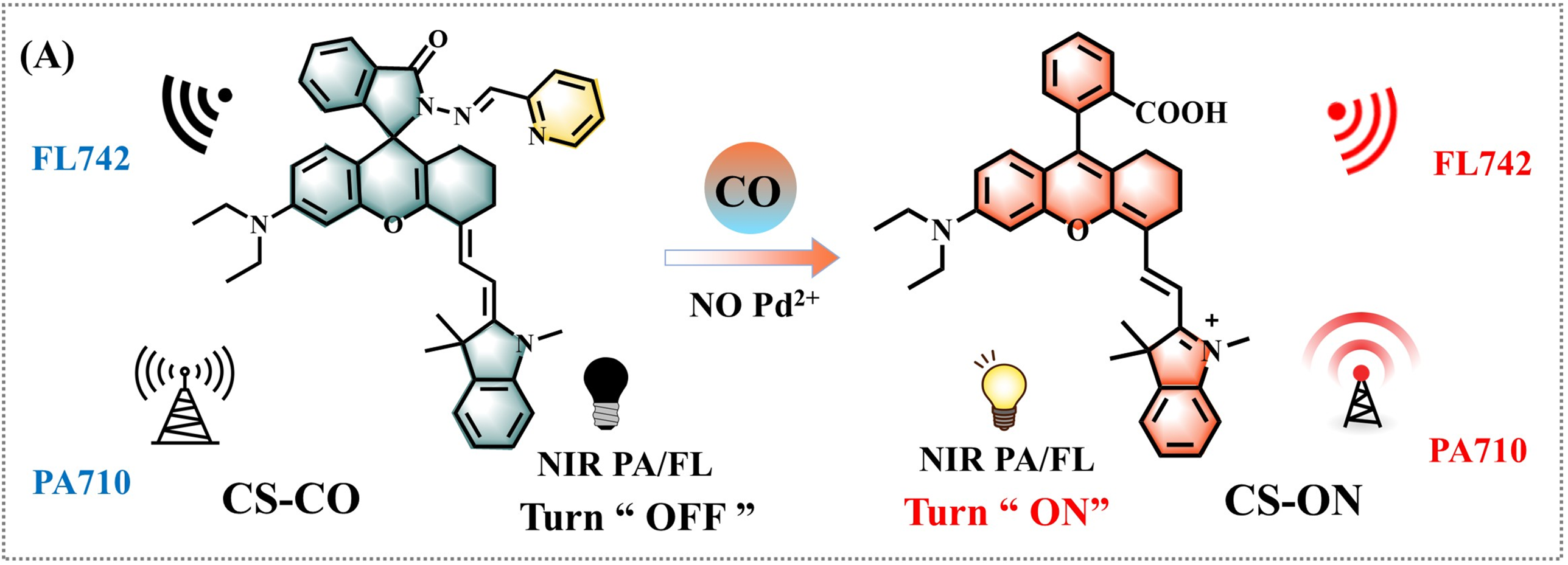


## **Figure S1** The possible response process of CS-CO for CO recognition.


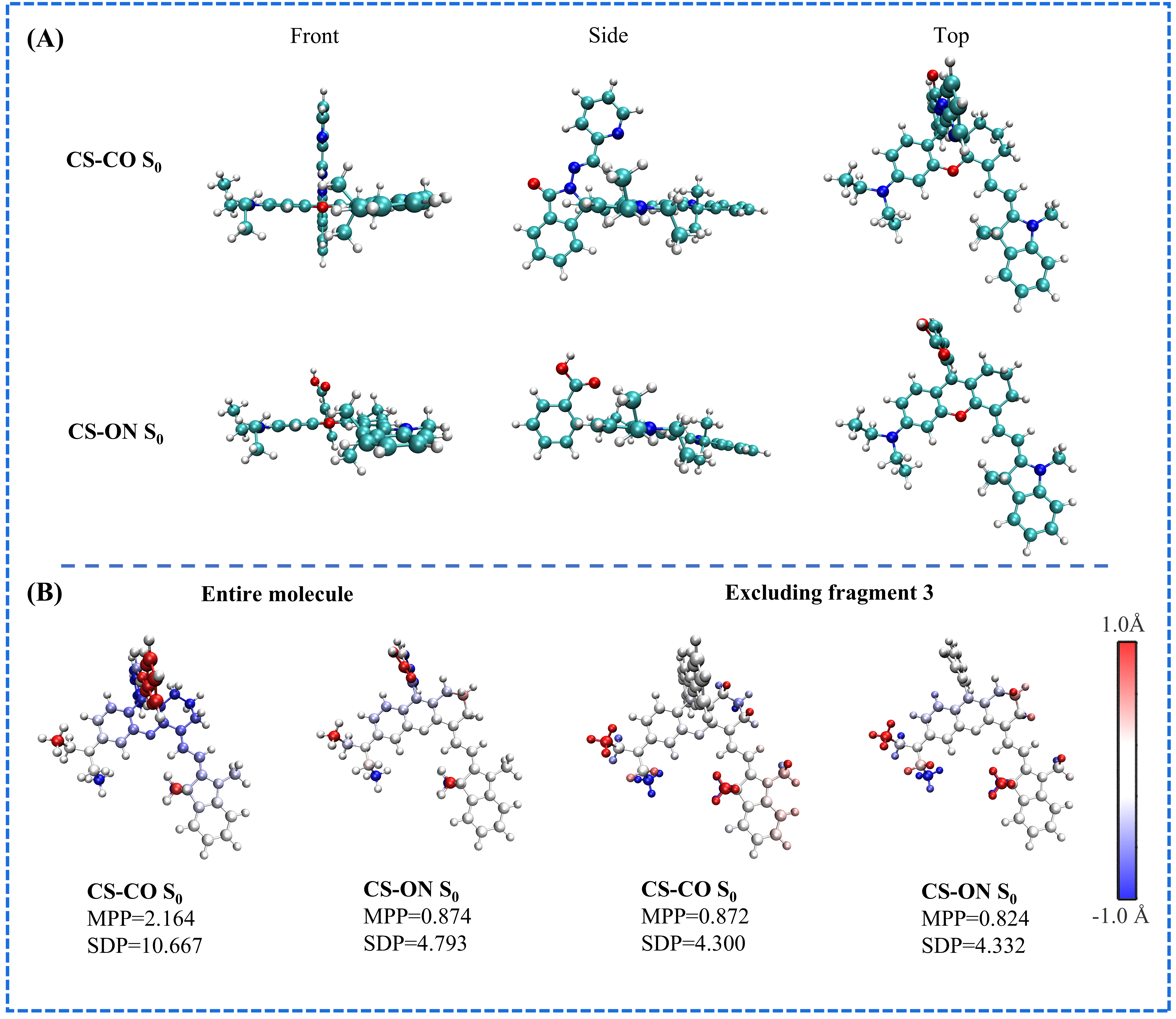


## **Figure S2** Geometric structures and planarity of CS-CO and CS-ON in S_0_ state. (A) Three-view diagram, (B) MPP and SDP.


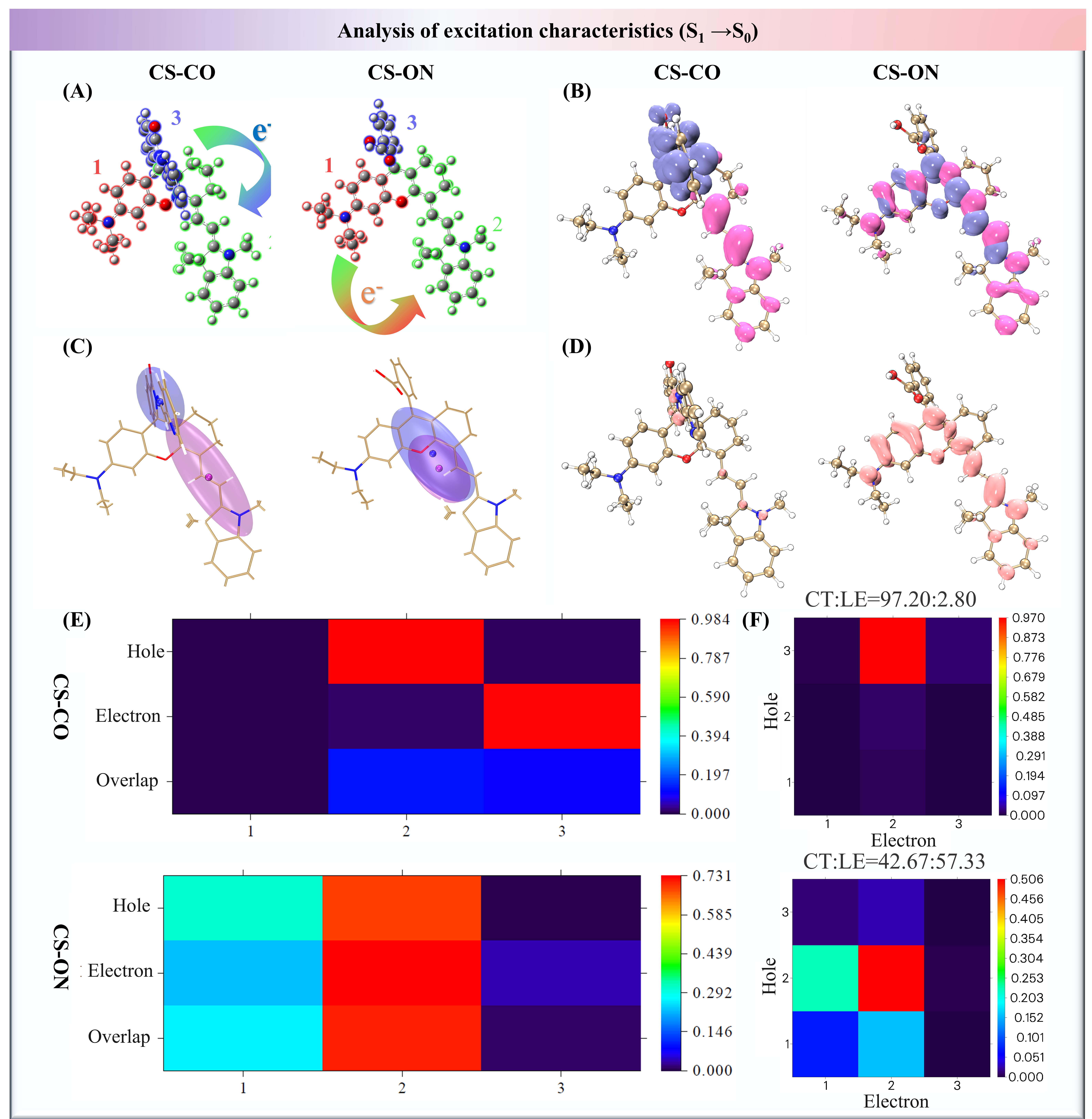


## **Figure S3** Excitation characterisation of CS-CO and CS-ON (S_1_→S_0_). (A) fragments and electron transfer pathway, (B) hole-electron distribution, (hole: bule, electron: purple) (C) hole-electron centroid, (hole centroid: bule, electron centroid: purple) (D) hole-electron overlap function (Sr), (E) contribution heat map of fragment to hole, electron, and their overlap, (F) interfragment charge transition matrix heat map.


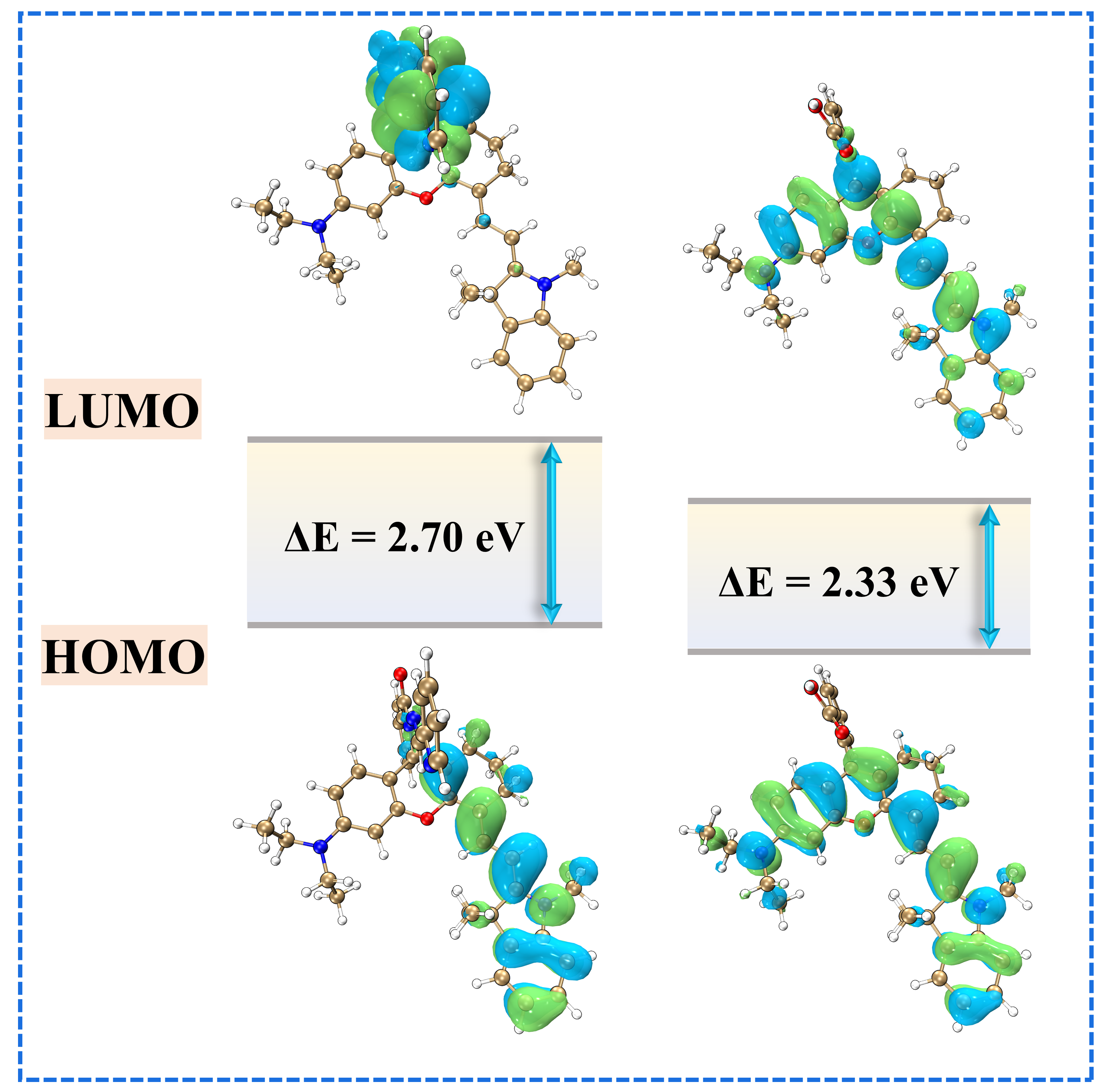


## **Figure S4** The Front molecular orbitals and energy gaps of CS-CO and CS-ON.


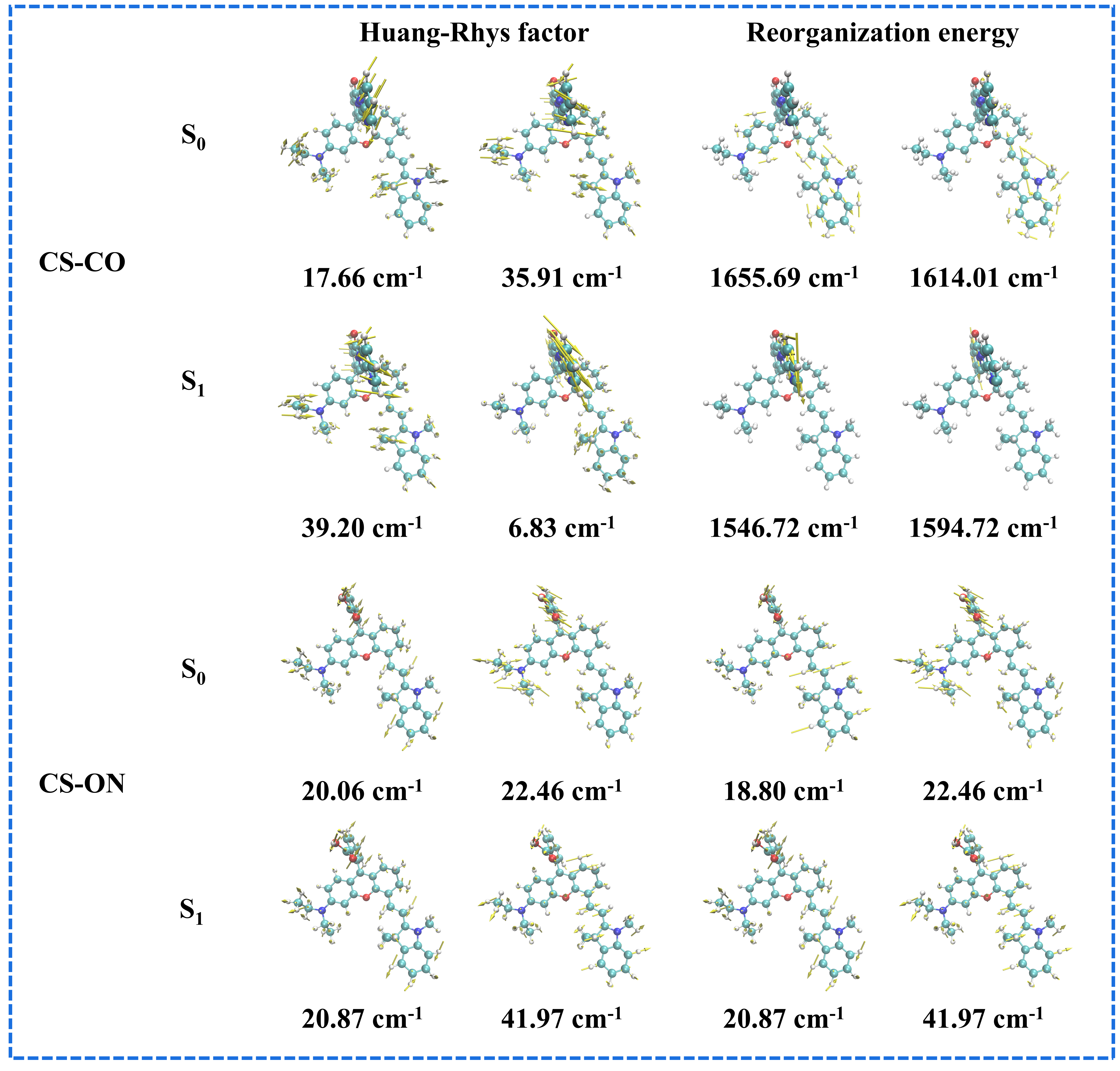


## **Figure S5** The representative normal modes of CS-CO and CS-ON in S_0_ and S_1_ states and their Huang-Rhys factors and reorganization energies.

## **Figure S6** The ^1^H NMR of CS-CO in CDCl_3_.

## **Figure S7** The ^13^C NMR of CS-CO in CDCl_3_.


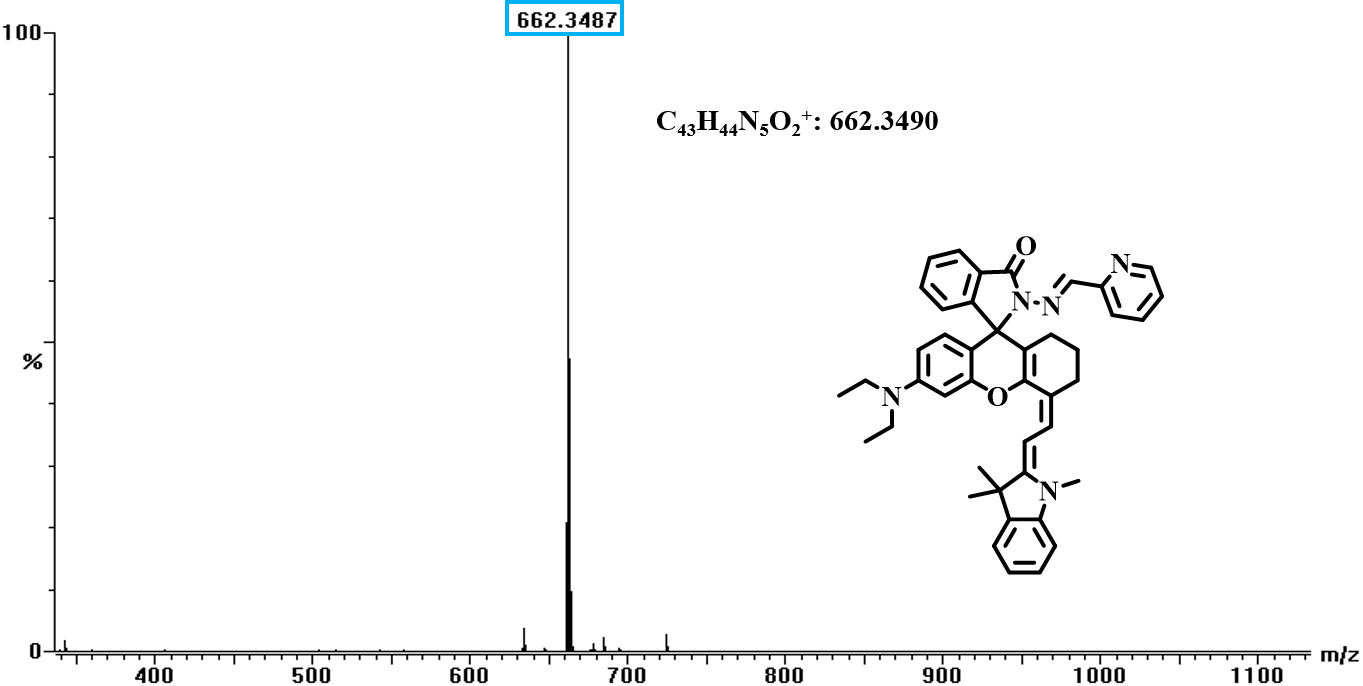


## **Figure S8** ESI-MS spectrum of CS-CO.


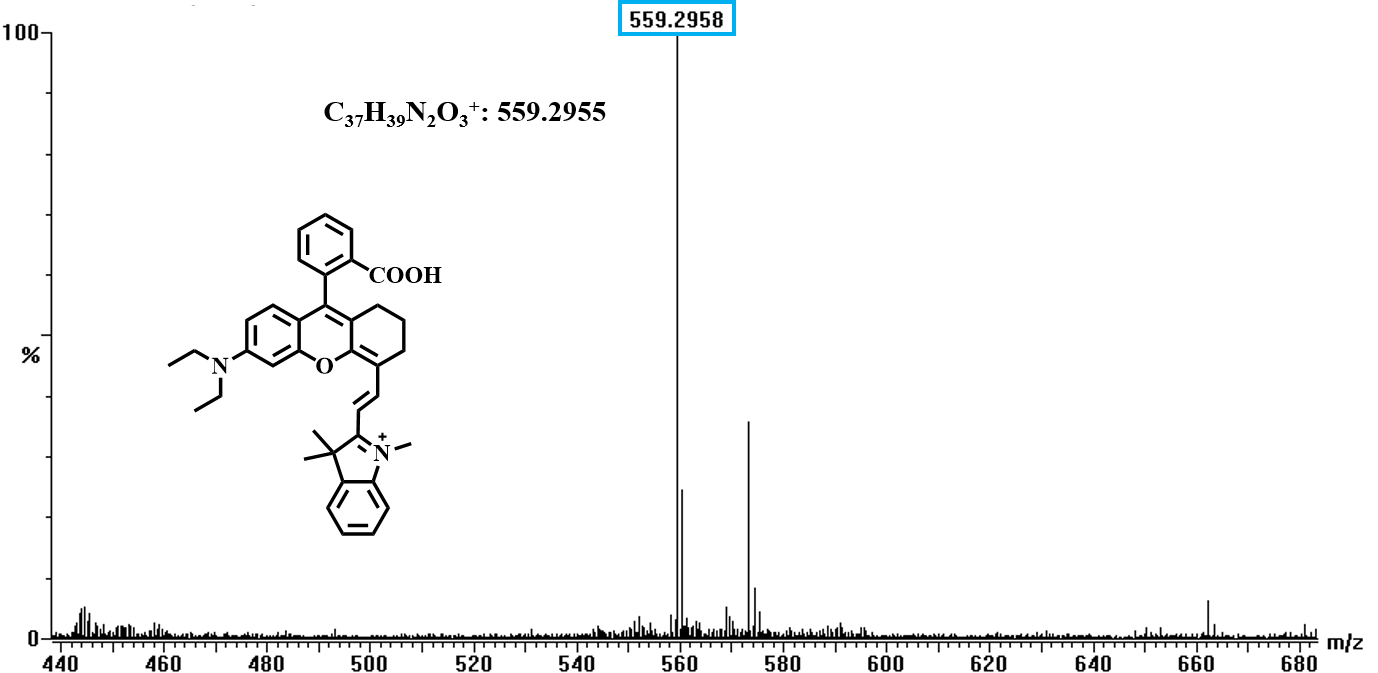


## **Figure S9** HRMS (ESI) spectra of probe CS-CO and CORM-3 after reaction.


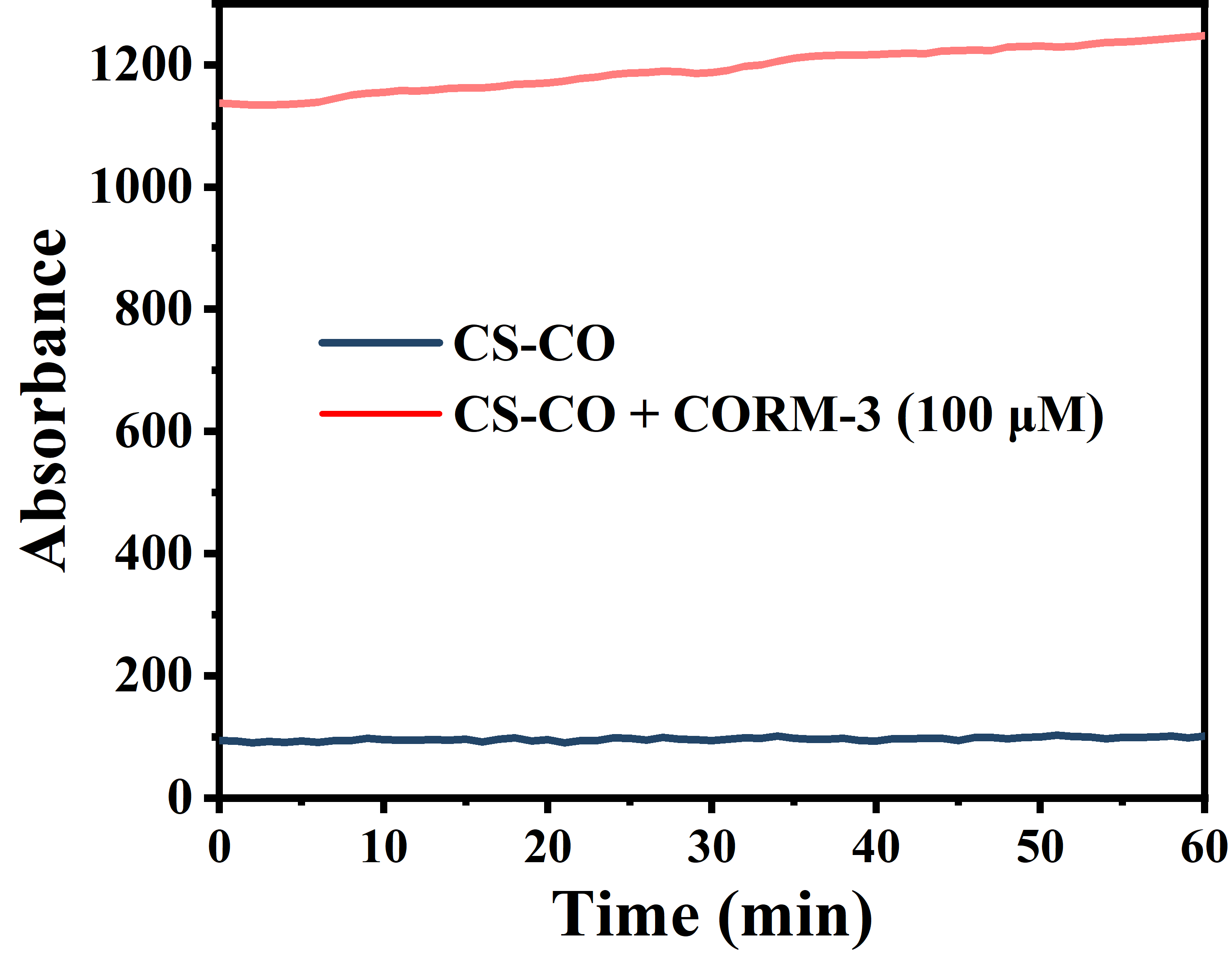


## **Figure S10** Photostability of CS-CO (10 μM) in the presence or absence of CORM-3 (100 μM).


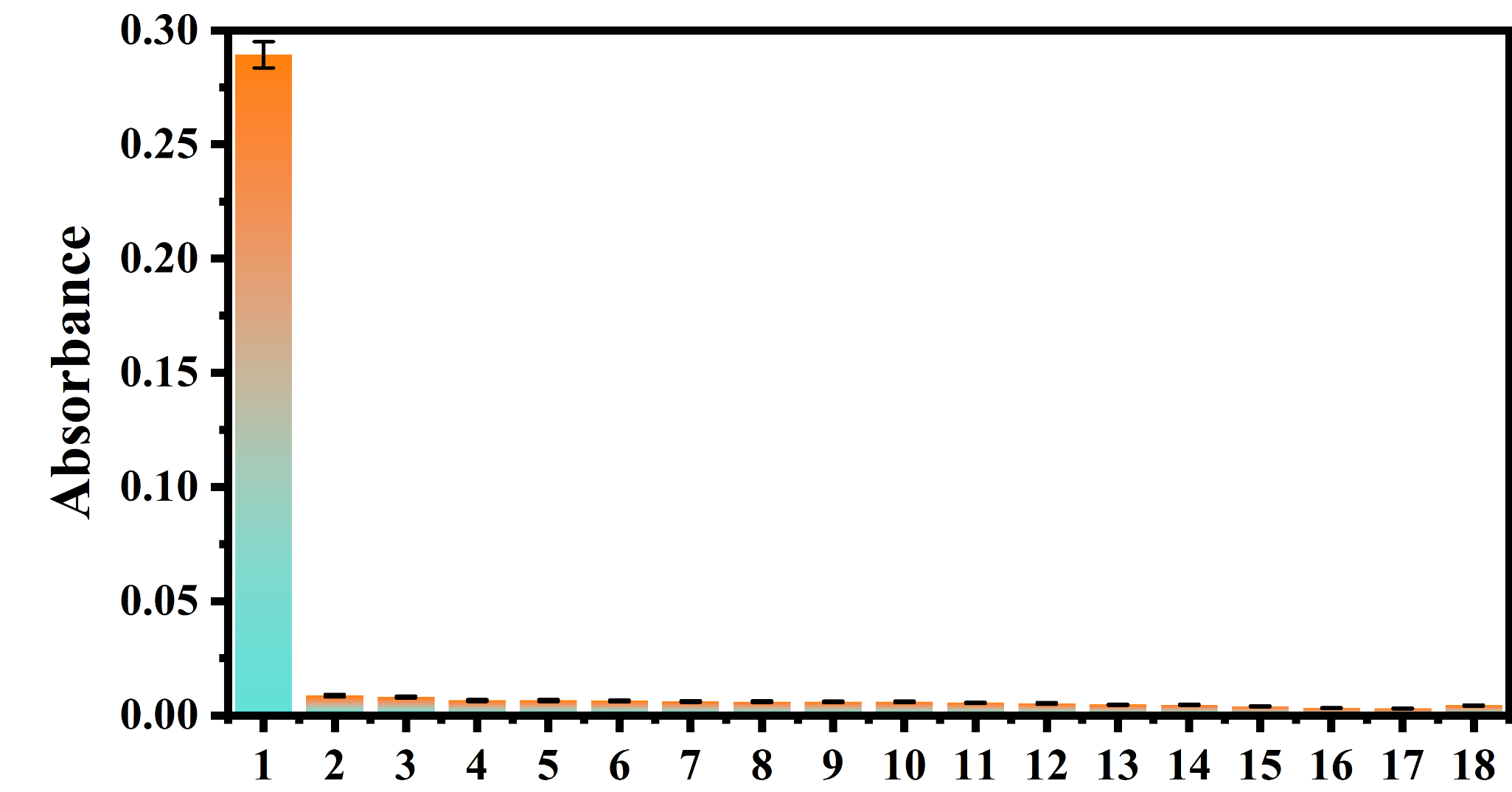


Figure S11 Absorption intensity of CS-CO (10 μM) after addition of various analytes. The analytes 1-18: 1. CORM-3(100 μM); 2. FeSO_4_ (100 μM); 3. Hg(NO_3_)_2_ (100 μM); 4. L-Cys (100 μM); 5. FA (100 μM); 6. CdCl_2_ (100 μM); 7. MnCl_2_ (100 μM); 8. CaCl_2_ (100 μM); 9. ZnCl_2_ (100 μM); 10. MgCl_2_ (100 μM); 11. NaNO_2_ (100 μM); 12. NaHS (100 μM); 13. Na_2_SO_3_ (100 μM); 14 KCl (100 μM); 15. NaSCN (100 μM); 16. L-Glutathione (500 μM); 17. DL-Hcy (500 μM); 18. PBS; Error bars represent standard deviation (± S.D.), n = 3.


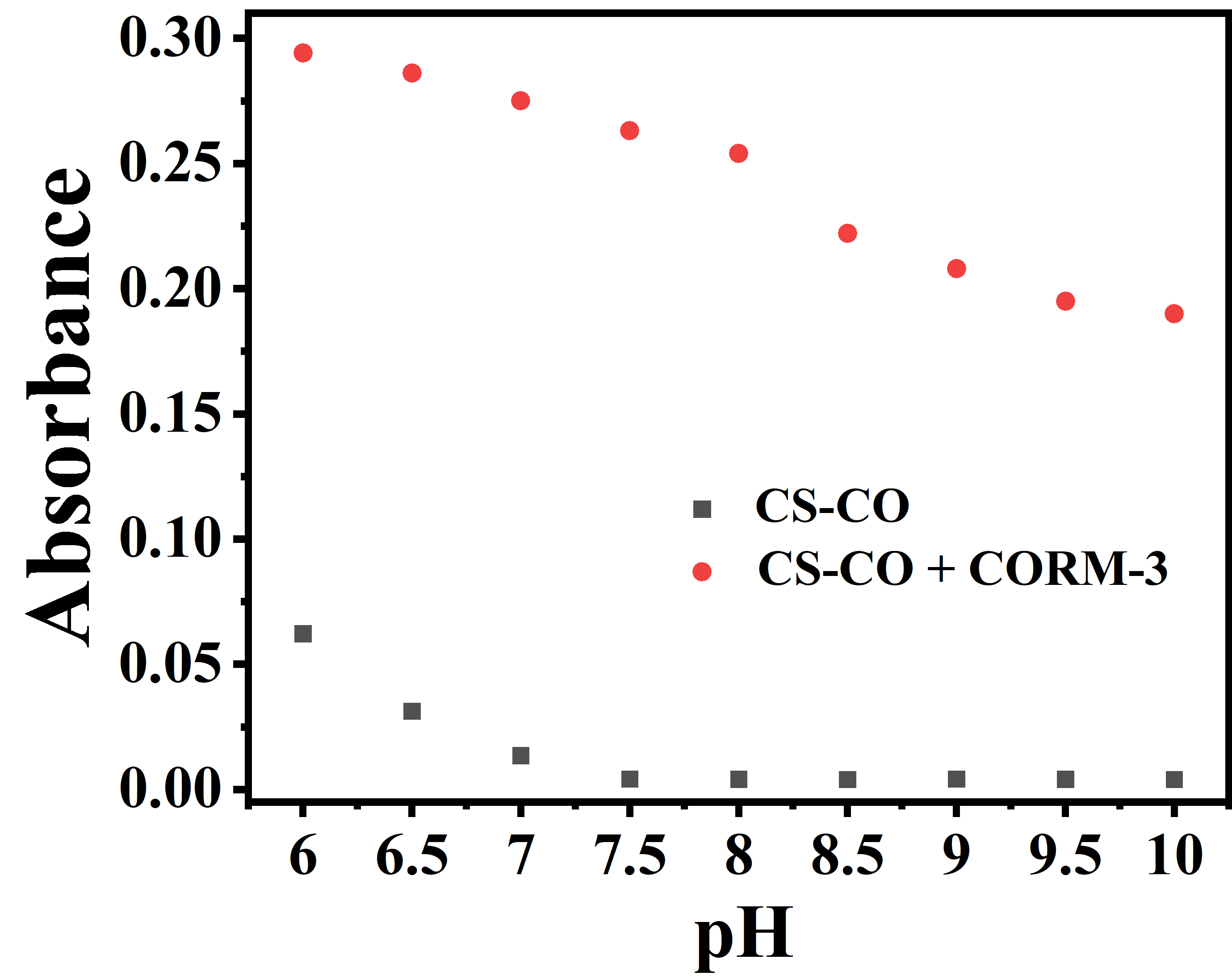


## **Figure S12** Absorption intensity changes of CS-CO at different pH values in the absence or presence of CORM-3.


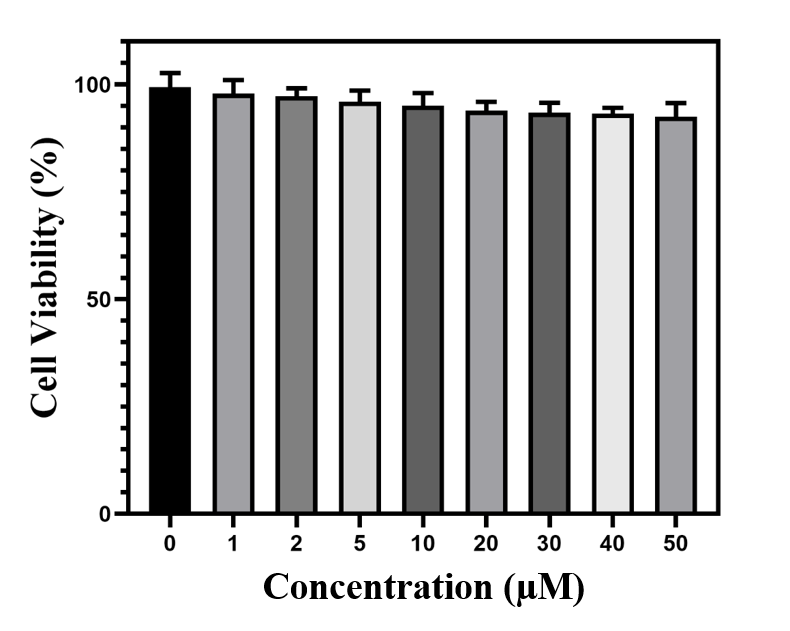


## **Figure S13** Survival rate of HeLa cells after 24 h of incubation with different concentrations of CS-CO by MTT assays.


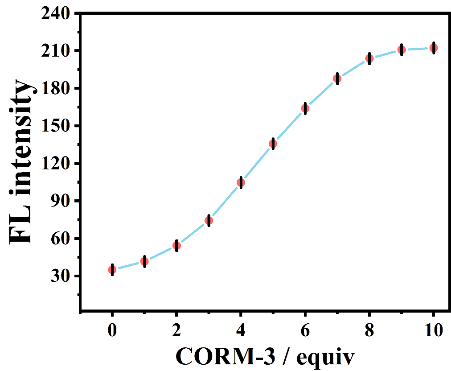


## **Figure S14** FL signal intensity of CS-CO after incubation with various concentrations of CORM-3.


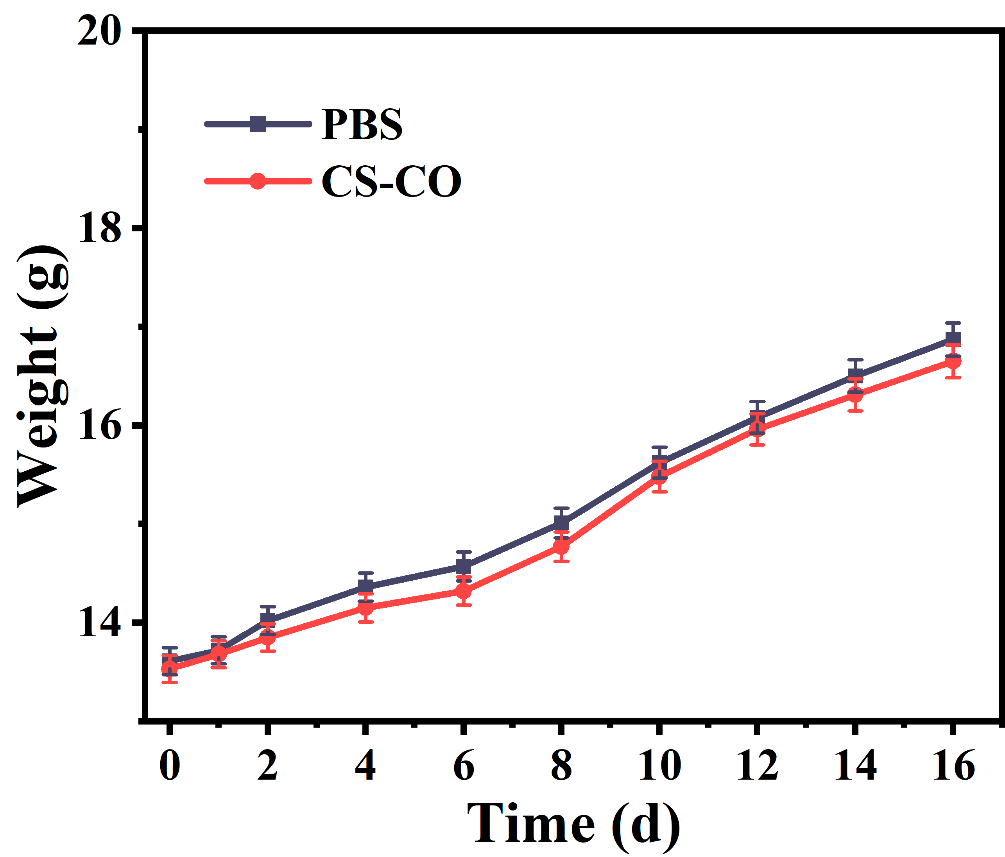


## **Figure S15** Body weight change of the mice during 16 days following the intravenous injection of PBS (control group) and CS-CO (100 μM) into the tail vein of the mice.

# Supplementary Tables

## **Table S1** Basic parameters of hole-electron analysis.

| Compound | Transition | D^a^ (Å) | Sr^b^ (a.u.) | t^c^ (Å) | Δσ^d^ (Å) | HDI^e^ | EDI^f^ | Type^g^ |
| --- | --- | --- | --- | --- | --- | --- | --- | --- |
| **CS-CO** | S_0_→S_1_ | 6.383 | 0.21626 | 3.882 | 0.084 | 7.72 | 7.02 | CT |
|  | S_1_→S_0_ | 6.109 | 0.16827 | 3.741 | 0.019 | 7.66 | 7.56 | CT |
| **CS-ON** | S_0_→S_1_ | 0.669 | 0.69073 | -2.070 | -0.680 | 6.42 | 7.35 | HLCT |
|  | S_1_→S_0_ | 1.132 | 0.68214 | -2.386 | -0.627 | 6.43 | 7.23 | HLCT |

^a^ distance between centroid of hole and electron.

^b^ overlap function between hole and electron distribution.

^c^ separation degree of hole and electron in CT direction.

^d^ distribution breadth difference index of electrons and holes.

^e^ hole delocalization index.

^f^ electron delocalization index.

^g^ CT=charge transfer, HLCT=hybrid local and charge transfer.

## **Table S2** Percentage contribution of each fragment to hole and electron.

| Compound | Transition | Fragment | Hole^a^ (%) | Electron^b^ (%) | Overlap^c^ (%) | Diff^d^ (%) |
| --- | --- | --- | --- | --- | --- | --- |
| **CS-CO** | S_0_→S_1_ | 1 | 0.37 | 0.67 | 0.50 | 0.30 |
|  |  | 2 | 98.23 | 2.95 | 17.04 | -95.27 |
|  |  | 3 | 1.40 | 96.38 | 11.62 | 94.98 |
|  | S_1_→S_0_ | 1 | 0.51 | 0.41 | 0.46 | -0.11 |
|  |  | 2 | 1.66 | 98.41 | 12.79 | 96.75 |
|  |  | 3 | 97.83 | 1.19 | 10.77 | -96.64 |
| **CS-ON** | S_0_→S_1_ | 1 | 35.57 | 22.27 | 28.15 | -13.29 |
|  |  | 2 | 64.09 | 75.64 | 69.63 | 11.55 |
|  |  | 3 | 0.34 | 2.09 | 0.84 | 1.75 |
|  | S_1_→S_0_ | 1 | 23.00 | 30.98 | 26.69 | 7.98 |
|  |  | 2 | 73.10 | 68.66 | 70.85 | -4.44 |
|  |  | 3 | 3.90 | 0.36 | 1.19 | -3.54 |

^a^ percentage contribution of each fragment to the hole.

^b^ percentage contribution of each fragment to electron.

^c^ percentage overlap of hole and electron in each fragment.

^d^ difference in percentage contribution of hole and electron from each fragment.

## **Table S3** Net amount (e) of electron transfer between fragments.

| Compound | Transition | 1→2 | 1→3 | 2→3 | CT^a^ (%) | LE^b^ (%) |
| --- | --- | --- | --- | --- | --- | --- |
| **CS-CO** | S_0_→S_1_ | -0.00647 | 0.00350 | 0.94625 | 95.745 | 4.255 |
|  | S_1_→S_0_ | 0.00498 | -0.00391 | -0.96249 | 97.203 | 2.797 |
| **CS-ON** | S_0_→S_1_ | 0.12628 | 0.00666 | 0.01080 | 43.591 | 56.409 |
|  | S_1_→S_0_ | -0.06854 | -0.01126 | -0.02417 | 42.670 | 57.330 |

^a^ intrinsic charge transfer percentage, CT=charge transfer.

^b^ intrinsic local excitation percentage, LE=local excitation

## **Table S4** The orbital transition type, transition orbital contribution and π component of CS-CO and CS-ON under the transition (S_0_→S_1_).

| Compound | Transition | Orbital transition | Contribution | π (HOMO) composition | π (LUMO) composition | Types |
| --- | --- | --- | --- | --- | --- | --- |
| **CS-CO** | S_0_→S_1_ | H→L | 98.7% | 90.920% | 86.192% | π→π* |
| **CS-ON** | S_0_→S_1_ | H→L | 99.5% | 93.285% | 78.394% | π→π* |

# **Optimized atomic coordinate information**

## **Table S5** Cartesian coordinates (Å) obtained after optimising **CS-CO** S_0_ at the B3LYP/defTZVP theoretical level in DCM, Energy = -2089.5722776 Hartree.

| **CS-CO** | Charge = 0 | Spin multiplicity = 1 | Method = DFT |
| --- | --- | --- | --- |
| Atom | x | y | z |
| C  N  N  C  C  C  C  O  C  C  C  C  C  C  O  C  C  C  C  C  C  C  C  C  C  C  C  C  C  C  C  N  C  C  C  C  N  C  C  C  C  C  C  C  C  C  C  C  C  N  H  H  H  H  H  H  H  H  H  H  H  H  H  H  H  H  H  H  H  H  H  H  H  H  H  H  H  H  H  H  H  H  H  H  H  H  H  H  H  H  H  H  H | 2.54733800  3.60509000  3.74115000  4.91333300  4.73858200  3.49388500  2.72712500  5.86803100  5.61757900  5.21096100  3.95409800  3.08126100  2.44626700  1.14287400  0.05923800  0.24724000  1.46903100  3.46679100  3.20879100  1.87461700  0.84593700  -0.99970600  -0.87306200  0.36154400  1.62200300  -2.18656300  -3.47786600  -4.65605300  -4.91071100  -6.42230800  -6.92998900  -5.87642700  -7.27342100  -8.65031100  -9.14472200  -8.29323700  1.59929900  0.23226100  2.65605200  -0.48049000  3.24633900  -4.24146400  -4.47862100  -6.01917000  1.04588900  1.96444300  3.15779500  3.38290600  2.40121600  1.24748500  1.71314700  6.58493200  5.86861500  3.65673700  2.10914900  4.49703800  4.04109600  -0.19558300  -1.76843000  -0.80862600  0.24941400  0.45175800  1.85749900  2.47671200  -2.13914300  -3.52390300  -6.88593300  -9.32930500  -10.21019800  -8.69673800  -0.33426700  0.27867000  3.43811900  2.22737000  -1.49307700  0.05673700  -0.54797500  3.69428400  4.01800200  2.47217200  -4.54015400  -3.15531500  -4.54717200  -4.78002900  -3.39909400  -4.95172800  -5.47880600  -7.06876900  -5.62937000  0.10544800  1.74859500  3.89938800  4.29361100 | -0.97877100  -1.41806500  -1.34200300  -1.81638600  -1.62000400  -1.05868700  -0.82265600  -2.28943000  -1.92480100  -1.65155600  -1.08844200  -0.78637500  0.64157700  1.11486300  0.28074000  -1.08617400  -1.65026000  1.58680500  2.93066000  3.41305600  2.46126500  -1.83318500  -3.33778900  -3.82105000  -3.14581200  -1.17925600  -1.78178900  -1.10833400  0.40885900  0.46153100  -0.84023200  -1.75016000  1.54517300  1.32458500  0.02466400  -1.08034400  4.74616700  5.22901600  5.75149200  5.49186300  5.96291200  1.08893500  1.07600400  -3.18899700  -0.62770500  -1.16241500  -1.67229600  -1.62817300  -1.07072200  -0.57701800  -0.52521000  -2.36278600  -1.87585800  -0.88501800  -0.35156100  1.25333600  3.60759300  2.74043400  -3.79606100  -3.68119100  -3.58260400  -4.90588100  -3.52940600  -3.40240100  -0.10201200  -2.86217400  2.55612200  2.16509700  -0.14039000  -2.08248000  4.51971700  6.15071400  5.48313400  6.68619000  5.85959500  6.24256600  4.58280500  5.04417700  6.73529100  6.27897100  2.13758700  1.04764900  0.60690800  2.12475300  1.03316200  0.58538800  -3.61765000  -3.44965700  -3.62946500  -0.22134600  -1.17759600  -2.09748100  -2.01217200 | 2.03239400  1.45752800  0.12229800  -0.46417000  -1.91496200  -2.16201400  -0.87023000  0.12737900  -2.94828700  -4.24890700  -4.49790100  -3.45506100  -0.65410000  -0.59359700  -0.68288900  -0.73945500  -0.82071800  -0.53498500  -0.36197000  -0.31183500  -0.43360800  -0.66789800  -0.64647400  -1.40558900  -0.87488300  -0.57125600  -0.44729600  -0.34273200  -0.32635300  -0.18462900  -0.13072700  -0.22524800  -0.10980000  0.02036800  0.07242500  -0.00212900  -0.14696800  0.01336100  -0.11010300  -1.31288300  1.28347600  0.88289300  -1.64563800  -0.20287200  5.28796900  6.18349900  5.67922600  4.31382700  3.48708000  3.96974700  1.51270800  -2.73940500  -5.07853900  -5.51875400  -3.64941200  -0.56713100  -0.25216200  -0.41286800  -1.06755600  0.39375900  -2.46742300  -1.32385200  0.12595700  -1.50516200  -0.57717800  -0.42665700  -0.15067400  0.08023100  0.17293100  0.04031000  0.61893500  0.59487500  -0.82125400  -0.47442200  -1.13364400  -1.89559000  -1.91190100  1.66454800  1.25678800  1.98529700  0.92646900  0.81664500  1.81215300  -1.64343500  -1.78174100  -2.49698600  0.64469200  -0.11195000  -1.12377900  5.64375300  7.24314700  6.34322800  3.87688500 |

## **Table S6** Cartesian coordinates (Å) obtained after optimising **CS-ON** S_0_ at the B3LYP/defTZVP theoretical level in DCM, Energy = -1769.2621675 Hartree.

| **CS-ON** | Charge = 0 | Spin multiplicity = 1 | Method = DFT |
| --- | --- | --- | --- |
| Atom | x | y | z |
| C  C  C  C  C  C  N  C  C  C  C  C  C  C  C  C  C  C  C  C  C  C  C  O  C  C  C  C  N  C  C  C  C  C  C  C  C  C  C  C  O  O  H  H  H  H  H  H  H  H  H  H  H  H  H  H  H  H  H  H  H  H  H  H  H  H  H  H  H  H  H  H  H  H  H  H  H  H  H  H  H | -8.33332700  -8.86626000  -8.04082300  -6.67082100  -6.12301100  -6.94889900  -5.62327600  -4.40901000  -4.61043700  -3.22933200  -1.95215400  -0.75652500  -0.70451300  0.64015400  1.78971000  1.72604500  0.46255600  -4.00112700  -4.06953300  -5.81869800  2.85241800  2.73849500  1.46040300  0.37085700  3.81190700  3.61630500  2.31244800  1.23049800  2.12427400  0.80900800  3.21883500  -0.03708600  3.97758300  5.14980200  4.19038300  6.40242600  6.70461400  5.75596900  4.51081900  4.82635400  3.70997300  5.92296000  -8.99473900  -9.93964800  -8.46928000  -6.53731700  -3.31204900  -1.87223700  -1.50307000  -0.89976600  0.67384100  0.75535900  2.75000600  1.74731100  -4.37662400  -2.91448200  -4.27845900  -4.49568400  -4.34461900  -2.98484800  -6.87782700  -5.43400600  -5.30638400  4.81377100  4.47478500  0.21253900  0.28776100  0.97750900  3.89336700  2.77874400  0.46707900  -0.22668600  -0.99787100  4.44011400  4.76303900  3.30537900  7.13309700  7.67529000  5.98320900  3.77584700  5.62920300 | 1.41081500  0.12466600  -1.00063800  -0.78597300  0.49503000  1.60224600  -1.72720400  -1.12700200  0.39397600  -1.85056000  -1.28735600  -1.98829400  -3.49883200  -4.02556300  -3.39813500  -1.89239300  -1.28362800  1.07567600  1.01334200  -3.16761700  -1.09618300  0.31747400  0.88026100  0.06794500  1.22805600  2.58099000  3.13747000  2.23562300  4.47908300  5.05451600  5.43411500  5.26437300  5.72974300  -1.86398900  -1.70541700  -2.40239700  -2.78855400  -2.63177900  -2.09163600  -1.49975600  -1.30969700  -1.40397700  2.26714200  -0.01056800  -1.99225400  2.60318200  -2.92791300  -0.21205100  -3.88614800  -3.87905900  -5.11173800  -3.78730600  -3.73231500  -3.73017300  0.61780300  1.01106400  2.13020700  0.51392000  2.06767600  0.94308600  -3.38379500  -3.62955900  -3.59172400  0.84530600  3.22943100  2.57078700  4.42330600  6.00862200  5.06901200  6.35113600  5.93528500  4.32076600  5.70851500  4.82707900  6.46455300  6.13362400  -2.52409400  -3.20961300  -2.92713100  -1.96003600  -1.19937700 | 0.07656200  0.11969900  0.12524300  0.08659500  0.04189300  0.03702300  0.08376400  0.03432500  0.00413000  0.01334600  -0.02946900  -0.06775100  -0.06048900  0.43407600  -0.34829300  -0.30007100  -0.14562900  1.24531100  -1.29977000  0.12564600  -0.38229400  -0.33864900  -0.16458700  -0.06988600  -0.44152600  -0.36784700  -0.18344100  -0.09044000  -0.10290900  0.18044000  -0.28598300  -1.07337900  1.00529100  0.40251300  -0.61212000  0.09136600  -1.20562200  -2.20986200  -1.91210200  1.80575200  2.23745700  2.58610200  0.07315500  0.14916100  0.15766800  0.00291300  0.02432700  -0.04648900  0.57405000  -1.06977300  0.33974800  1.49527500  0.04365700  -1.39141800  2.16068500  1.24827400  1.24950600  -2.17015400  -1.33926700  -1.35911600  0.20777400  -0.78435800  0.98952800  -0.57910300  -0.43668900  0.02404300  0.90016000  0.67974800  -1.05943900  -0.67794500  -1.77091000  -1.58632500  -0.80677200  1.40656100  0.81934000  1.76424500  0.87738500  -1.43120200  -3.22595000  -2.69530300  3.48832000 |

## **Table S7** Cartesian coordinates (Å) obtained after optimising **CS-CO** S_1_ at the B3LYP/defTZVP theoretical level in DCM, Energy = -2089.49907662 Hartree.

| **CS-CO** | Charge = 0 | Spin multiplicity = 1 | Method = TD-DFT |
| --- | --- | --- | --- |
| Atom | x | y | z |
| C  N  N  C  C  C  C  O  C  C  C  C  C  C  O  C  C  C  C  C  C  C  C  C  C  C  C  C  C  C  C  N  C  C  C  C  N  C  C  C  C  C  C  C  C  C  C  C  C  N  H  H  H  H  H  H  H  H  H  H  H  H  H  H  H  H  H  H  H  H  H  H  H  H  H  H  H  H  H  H  H  H  H  H  H  H  H  H  H  H  H  H  H | 2.39763200  3.57712700  3.74241100  4.93306500  4.80279400  3.55598100  2.77443300  5.89363900  5.71026000  5.32994200  4.07500300  3.16959600  2.46792800  1.17297500  0.11110700  0.28821800  1.52091300  3.46015800  3.17748000  1.85084200  0.84959600  -0.92612400  -0.83216400  0.48076100  1.66550600  -2.13490600  -3.37811500  -4.58040400  -4.82517000  -6.32711600  -6.82906600  -5.75386300  -7.18566700  -8.55024600  -9.03666300  -8.17983400  1.55248600  0.17502900  2.59142000  -0.35623800  2.97599800  -4.11740400  -4.42735500  -5.90474200  0.64808100  1.57257600  2.86830300  3.17071600  2.16716500  0.90445600  1.54503200  6.68170600  6.01682500  3.80070200  2.19711500  4.48164000  3.98447300  -0.18488700  -1.67638400  -0.92392700  0.50009400  0.55482500  1.75512600  2.60256400  -2.07600300  -3.42740900  -6.81427800  -9.23780500  -10.09696600  -8.56823300  -0.46492400  0.14074400  3.46749200  2.21815100  -1.38454900  0.25194500  -0.34132400  3.36440800  3.74363400  2.10938000  -4.42254500  -3.03466400  -4.39061400  -4.73954100  -3.35123200  -4.91542800  -5.30749400  -6.94606200  -5.58964300  -0.36512000  1.29544300  3.62631900  4.15817400 | -0.88411600  -1.24489400  -1.34403500  -1.82686900  -1.75129200  -1.22911100  -0.90216900  -2.23721800  -2.11630300  -1.95156900  -1.43459200  -1.06709300  0.57023400  1.03800800  0.18122600  -1.16888600  -1.73773200  1.52627400  2.87602300  3.35042100  2.38489300  -1.91489400  -3.40866600  -3.96847500  -3.21260800  -1.23149500  -1.82663000  -1.12995400  0.37914700  0.43974100  -0.85253100  -1.76309300  1.52161900  1.28630200  -0.01342200  -1.11141300  4.68821500  5.16527900  5.70312500  5.36073000  5.98997400  1.11366300  0.96699100  -3.21004800  -0.26076100  -0.49077500  -0.87709700  -1.01150600  -0.75972700  -0.38190500  -0.66179100  -2.51625100  -2.22806300  -1.31659500  -0.66241200  1.19520600  3.56491200  2.65503700  -3.88609700  -3.65170500  -3.87081800  -5.03050000  -3.42000900  -3.54176000  -0.15937000  -2.90273300  2.53268600  2.12088900  -0.17699100  -2.11383000  4.47950400  6.11315800  5.40577600  6.61455400  5.72769300  6.08850300  4.42468300  5.09539500  6.76557200  6.33621900  2.15995200  1.07503000  0.67656700  2.01018500  0.92836300  0.42566500  -3.58272100  -3.44593600  -3.69059300  0.04091900  -0.37303900  -1.06725900  -1.30621400 | 2.13156100  1.64772300  0.31405100  -0.21095200  -1.66401500  -2.00514500  -0.73933600  0.45552200  -2.66020500  -3.98963100  -4.32279900  -3.31828000  -0.63483700  -0.78695400  -0.99538900  -0.82494200  -0.69908800  -0.42020000  -0.36280000  -0.53046600  -0.74703600  -0.71173700  -0.56455600  -1.10229500  -0.51338800  -0.65607400  -0.47434000  -0.36870100  -0.42774100  -0.24643500  -0.10592800  -0.18154100  -0.20604600  -0.02429600  0.11304700  0.07436000  -0.47595700  -0.52095900  -0.33939700  -1.94064500  1.11160700  0.72976700  -1.79714700  -0.06984700  5.20865900  6.22467600  5.84132500  4.50720300  3.52892900  3.90947200  1.50378800  -2.39984300  -4.78001800  -5.36297400  -3.57034100  -0.27929400  -0.16952400  -0.88819800  -1.06265200  0.50129300  -2.19096100  -0.86754300  0.56092700  -0.96443400  -0.73840700  -0.39581200  -0.31223100  0.00993700  0.25222900  0.18088600  0.03690500  0.01782900  -0.91680000  -0.80882200  -1.91455200  -2.48115800  -2.50048900  1.60021100  1.15561400  1.67809500  0.72781900  0.62775300  1.69013700  -1.84229300  -1.95377300  -2.60756600  0.76080800  0.11364300  -0.99501900  5.46251100  7.26295300  6.59240100  4.18020100 |

## **Table S8** Cartesian coordinates (Å) obtained after optimising **CS-ON** S_1_ at the B3LYP/defTZVP theoretical level in DCM, Energy = -1769.19409136 Hartree.

| **CS-ON** | Charge = 0 | Spin multiplicity = 1 | Method = TD-DFT |
| --- | --- | --- | --- |
| Atom | x | y | z |
| C  C  C  C  C  C  N  C  C  C  C  C  C  C  C  C  C  C  C  C  C  C  C  O  C  C  C  C  N  C  C  C  C  C  C  C  C  C  C  C  O  O  H  H  H  H  H  H  H  H  H  H  H  H  H  H  H  H  H  H  H  H  H  H  H  H  H  H  H  H  H  H  H  H  H  H  H  H  H  H  H | -8.39126700  -8.92368300  -8.10139000  -6.72452400  -6.17834600  -7.00676800  -5.68984700  -4.46183300  -4.66717300  -3.28442300  -1.99141700  -0.81320700  -0.80277800  0.51067700  1.69872400  1.67703300  0.43867300  -4.04886800  -4.13279100  -5.88071300  2.84974700  2.75612800  1.48602400  0.36442000  3.84889900  3.69339800  2.40800400  1.30186200  2.25053700  0.95282600  3.36457800  0.10818100  4.13750300  5.16706000  4.17014900  6.42140200  6.69250300  5.70900700  4.46583800  4.87814900  3.78070400  5.99465300  -9.05471000  -9.99699700  -8.52925200  -6.59831500  -3.37685200  -1.88296700  -1.63567500  -0.96486500  0.51300300  0.60316800  2.63894500  1.68160700  -4.42412200  -2.96291100  -4.31751800  -4.57220800  -4.39625100  -3.04954100  -6.93933000  -5.48471500  -5.37292700  4.83908000  4.56888300  0.29401200  0.41893400  1.15083700  4.02491600  2.94111300  0.62797500  -0.11175900  -0.83660200  4.58332300  4.93632800  3.47991200  7.17639600  7.66419900  5.91111700  3.71219700  5.73544700 | 1.46599300  0.17560200  -0.94674700  -0.73280600  0.55811000  1.66106400  -1.66217700  -1.05278200  0.46259000  -1.77594200  -1.23159800  -1.97324400  -3.48032600  -4.04428000  -3.45883700  -1.95267200  -1.31539600  1.13805500  1.10138400  -3.10199500  -1.19669900  0.22399200  0.82647500  0.05992600  1.11081100  2.47257900  3.06244700  2.18734400  4.40983200  5.02138700  5.33706600  5.24607500  5.63146900  -1.79971300  -1.84722000  -2.36775800  -3.00170400  -3.06178200  -2.48428500  -1.21614800  -1.12748700  -0.79566700  2.32058800  0.04119200  -1.93871000  2.66347800  -2.85323600  -0.15962000  -3.85849700  -3.83766900  -5.13154600  -3.80237400  -3.80779900  -3.81205700  0.67946400  1.06333900  2.19499500  0.62115200  2.15946700  1.01886900  -3.32428600  -3.55309800  -3.53306200  0.70197900  3.09529400  2.55338300  4.40743300  5.97408200  4.94415200  6.25848900  5.89679500  4.30392600  5.72054100  4.72367400  6.34659600  6.06011500  -2.32470400  -3.44605100  -3.54953700  -2.50877600  -0.47879000 | 0.13370200  0.16509400  0.15275600  0.10798800  0.07505900  0.08820400  0.08820800  0.04030500  0.02720800  0.00896600  -0.03591500  -0.08433500  -0.08714300  0.44747500  -0.30793300  -0.30006600  -0.16320400  1.26959100  -1.27239100  0.11443300  -0.39022900  -0.36694300  -0.20491000  -0.10812500  -0.49767300  -0.44686100  -0.25860400  -0.14927100  -0.19235600  0.09499100  -0.39714400  -1.15860000  0.88722700  0.44802800  -0.54644700  0.20720200  -0.99644000  -1.97795000  -1.75330600  1.78399100  2.28988300  2.41744200  0.14438000  0.19952200  0.17657300  0.06362500  0.01342500  -0.04729600  0.50580800  -1.11162200  0.35985200  1.50991600  0.11863400  -1.34538100  2.18460500  1.26591200  1.27803400  -2.14685000  -1.29080800  -1.34086900  0.18441600  -0.79673900  0.97826800  -0.64211200  -0.53785900  -0.03801300  0.81935800  0.58578100  -1.16862000  -0.79670200  -1.86334800  -1.66153000  -0.88781300  1.29516100  0.68361100  1.64503800  0.97889100  -1.16642100  -2.92291100  -2.52931800  3.29770100 |

# References

[1] L. Yuan, W. Lin, Y. Yang, H. Chen, *J. Am. Chem. Soc.* **2012**, *134*, 1200–1211.
